# Novel Autotaxin Inhibitor ATX-1d Significantly Enhances Potency of Paclitaxel—An In Silico and In Vitro Study

**DOI:** 10.3390/molecules29184285

**Published:** 2024-09-10

**Authors:** Prateek Rai, Christopher J. Clark, Carl B. Womack, Curtis Dearing, Joshua Thammathong, Derek D. Norman, Gábor J. Tigyi, Subhabrata Sen, Kevin Bicker, April M. Weissmiller, Souvik Banerjee

**Affiliations:** 1 Molecular Biosciences, Middle Tennessee State University, Murfreesboro, TN 37132, USA; pr3x@mtmail.mtsu.edu (P.R.); cjc8g@mtmail.mtsu.edu (C.J.C.); kevin.bicker@mtsu.edu (K.B.); april.weissmiller@mtsu.edu (A.M.W.); 2 Department of Chemistry, Middle Tennessee State University, Murfreesboro, TN 37132, USA; dearingcurtis@gmail.com (C.D.); jt8p@mtmail.mtsu.edu (J.T.); 3 Department of Biology, Middle Tennessee State University, Murfreesboro, TN 37132, USA; cbw4@mtmail.mtsu.edu; 4 Department of Physiology, University of Tennessee Health Science Center, Memphis, TN 38163, USA; dnorman7@uthsc.edu (D.D.N.); gtigyi@uthsc.edu (G.J.T.); 5 Department of Chemistry, School of Natural Sciences, Shiv Nadar Institution of Eminence Deemed to be University, Dadri 201314, UP, India; subhabrata.sen@snu.edu.in

**Keywords:** autotaxin, molecular simulations, SAPT, QM/MM-GBSA, breast cancer, melanoma, combination therapy

## Abstract

The development of drug resistance in cancer cells poses a significant challenge for treatment, with nearly 90% of cancer-related deaths attributed to it. Over 50% of ovarian cancer patients and 30–40% of breast cancer patients exhibit resistance to therapies such as Taxol. Previous literature has shown that cytotoxic cancer therapies and ionizing radiation damage tumors, prompting cancer cells to exploit the autotaxin (ATX)–lysophosphatidic acid (LPA)–lysophosphatidic acid receptor (LPAR) signaling axis to enhance survival pathways, thus reducing treatment efficacy. Therefore, targeting this signaling axis has become a crucial strategy to overcome some forms of cancer resistance. Addressing this challenge, we identified and assessed ATX-1d, a novel compound targeting ATX, through computational methods and in vitro assays. ATX-1d exhibited an IC_50_ of 1.8 ± 0.3 μM for ATX inhibition and demonstrated a significant binding affinity for ATX, as confirmed by MM-GBSA, QM/MM-GBSA, and SAPT in silico methods. ATX-1d significantly amplified the potency of paclitaxel, increasing its effectiveness tenfold in 4T1 murine breast carcinoma cells and fourfold in A375 human melanoma cells without inducing cytotoxic effects as a single agent.

## 1. Introduction

**Figure 1:**
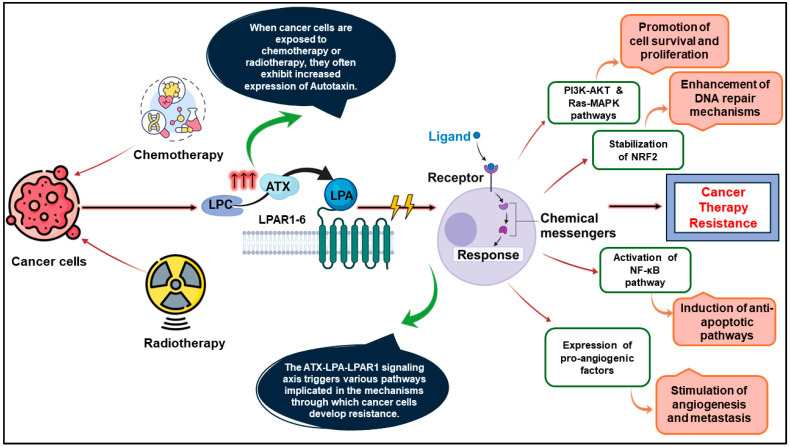
ATX-LPA-LPAR signaling axis in cancer therapeutic resistance.

The autotaxin–lysophosphatidic acid–lysophosphatidic acid receptors (ATX-LPA-LPAR) signaling axis is pivotal in numerous physiological and pathological processes within the body. Autotaxin (ATX) is central to this complex system, as it converts lysophosphatidylcholine (LPC) into lysophosphatidic acid (LPA), thereby precisely regulating LPA levels within the body. ATX was first identified in conditioned media from A2058 melanoma cells as an autocrine motility factor. It generates LPA, activating six *bona fide* G protein-coupled receptors (GPCR) designated as LPAR1-6. This interface initiates a signaling process that leads to cell proliferation, migration, survival, and inflammation [1,2,3,4]. Dysregulation of the ATX-LPA-LPAR axis has been linked to several diseases, including cancer, fibrosis, autoimmune disorders, neurodegenerative diseases, and cardiovascular disorders [5,6]. Notably, in cancer biology, this axis regulates vital processes such as malignant transformation, invasion, and metastasis [7]. The biggest challenges for effective cancer treatment have been identified as (i) resistance to conventional chemo- and radiation therapy, (ii) the intricate connections within the tumor microenvironment (TME), and evasion of tumor immunity [8,9,10,11,12]. Cytotoxic cancer therapies and ionizing radiation damage the tumor, initiating a self-repair mechanism that is profoundly affected by the ATX-LPA-LPAR axis. Consequently, cancer cells leverage this mechanism to upregulate survival pathways, thereby diminishing the efficacy of subsequent therapy [13,14,15]. The ATX-LPA-LPAR signaling axis has been implicated in chemotherapeutic and radiotherapeutic resistance through multiple mechanisms (Figure 1). These include (i) facilitation of cell survival and proliferation via the PI3K-AKT and RAS-MAPK pathways [16], (ii) augmentation of DNA repair mechanisms [15], (iii) induction of anti-apoptotic processes mediated by the NF-κB pathway, thereby impeding apoptosis [17], (iv) promotion of angiogenesis [18], and (v) modulation of the tumor microenvironment by fostering inflammation and immune suppression [19]. Emerging evidence suggests that the anticancer efficacy of taxanes, doxorubicin, tamoxifen, and sunitinib is intricately linked to the ATX-LPA-LPAR axis [13,20,21,22]. This relationship is partly due to the signaling ability of LPA to reinforce the stability of the transcription factor NRF2. The overexpression of ATX, alongside LPAR1 and LPAR2, has been documented across various tumor types, encompassing including ovarian cancer, osteocarcinoma, metastatic melanoma, neuroblastoma, breast cancer, pancreatic cancer, prostate cancer, and hepatocellular carcinoma [23]. Targeting the ATX-LPA-LPAR signaling axis with inhibitors holds promise for alleviating treatment resistance in patients whose cancer cells resist conventional anticancer therapies. Previous research has highlighted the role of the ATX-LPA-LPAR signaling axis in overcoming cancer resistance when inhibited in combination with chemotherapeutic agents. Li et al. (2011) revealed an intrinsic HDAC inhibitor (HDACi)-resistant mechanism in cancer cells, suggesting that inhibiting the ATX-LPA axis could enhance the efficacy of HDACi-based therapies [24]. Similarly, Long et al. (2023) demonstrated that the combined inhibition of EZH2 and the ATX-LPA-LPAR2 axis exerts synergistic antitumor effects in colon cancer cells [25]. In a significant advancement, the FDA has approved the ATX inhibitor IOA-289 as a potential therapeutic option for patients with pancreatic cancer. IOA-289 was evaluated for metastatic pancreatic cancer in combination with the standard care regimen of nab-paclitaxel and gemcitabine [26].

ATX features a tripartite binding site comprising a catalytic site with two zinc ions, a hydrophobic pocket, and a tunnel. Researchers have explored lipid-like and non-lipid ATX inhibitors, leveraging different mechanisms and binding modes to achieve effective inhibition [27]. Early efforts focused on lipid-like ATX inhibitors that mimic natural substrates like LPC. However, these inhibitors faced limitations in preclinical development due to high partition coefficients (logP > 5), impacting their pharmacokinetic profiles [28]. Despite these challenges, structural insights into ATX, mainly through X-ray cocrystal structures, have facilitated the design of more potent inhibitors [29]. A significant advancement came with the discovery of potent non-acidic inhibitors, such as compound 3, which exhibited an ATX IC_50_ of 2.2 nM [30]. These inhibitors demonstrated the potential for high efficacy without relying on acidic moieties, typically required to interact with the Zn^2+^ ions at the active site of the enzyme. Recent research has also explored hybrid inhibitors that combine features of multiple binding modes. These compounds, like GLPG1690, aim to enhance potency and specificity by targeting both the hydrophobic pocket and the tunnel of ATX. Zhang et al. reviewed the recent development of ATX inhibitors [31].

In this study, we report the discovery of a novel hit compound, ATX-1d, identified through in silico and in vitro methods, which enhances the potency of paclitaxel (PTX) in breast cancer and melanoma cell lines. Our approach began with a small library of 60 unique compounds subjected to molecular docking against ATX (PDB ID: 6W35), followed by an ATX enzyme inhibition assay. The library contained compounds from six distinct chemical families: itaconimides, imide-fused pyrroles, 4,6-dioxo-hexahydro-1H-furo [3,4-c]pyrrole derivatives, phenyloxazoli-din-3-yl)pentane-1,5-dione derivatives, spiro[cyclopropane-1,3′-indolin]-2′-one derivatives, and glycine betaine derivatives. To our knowledge, none of the compounds from these families were tested before for their biological activity against autotaxin. This process identified one compound, ATX-1d, that inhibited the ATX hydrolysis of the LPC-like fluorogenic substrate FS-3 by over 50% at a concentration of 10 μM. We further explored the potential interactions of ATX-1d using computational analyses, including molecular dynamics (MD) simulations, molecular mechanics/generalized Born surface area (MM-GBSA), and QM/MM-GBSA. Quantum chemical calculations employing symmetry-adapted perturbation theory (SAPT) and conceptual DFT were conducted to evaluate the strength of intermolecular interactions between the protein and the ligand and to explore the reactive properties of the compound. The computational analyses demonstrated that ATX-1d exhibits an effective binding affinity to ATX. Subsequently, ATX-1d was tested in combination with PTX through an in vitro synergistic assay against A375 melanoma cells and 4T1 breast carcinoma cells, demonstrating significant enhancement of the antitumor activity of PTX.

## 2. Methods

**Figure 2:**
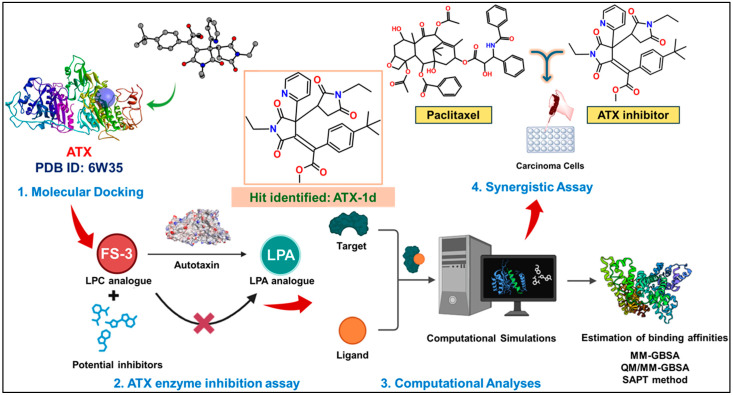
Overview of this study.

The methodology followed in this study is demonstrated in Figure 2.

## 3. Computational Details

### 3.1. Molecular Docking

The three-dimensional structures of 60 compounds were generated using *LigandScout* (version 4.4.3) modeling software through MMFF94 energy minimization [32,33]. The 3D structure of the protein target ATX (PDB ID: 6W35) was obtained from the RCSB Protein Data Bank (PDB) [34,35]. The energy minimization of the protein structures was done using the GROMOS96 force field within the *Swiss PDB-Viewer 4.1* [36] program to remove bad contacts and unfavorable torsion angles. Molecular docking was conducted using *PLANTS* software, version 1.2 [37], which integrates an ant colony optimization algorithm with an empirical scoring function for predicting and scoring binding poses of ligands within protein structures. For each compound, 50 binding poses were calculated and scored using the ChemPLP scoring function at a speed setting of 1. All other PLANTS settings were kept at their default values. The results, represented by the best binding affinity, were visualized using *Biovia Discovery Studio* (https://www.3ds.com/about, accessed on 5 September 2024) [38] to illustrate the interactions between the ligands and the targets. The docking procedure was validated by re-docking the native ligands to ensure alignment with their crystal structure poses. The root-mean-square deviations (RMSDs) between the docked and crystal poses were calculated using the *DockRMSD* (https://zhanggroup.org/DockRMSD/, accessed on 5 September 2024) web server to confirm the accuracy [39].

### 3.2. Molecular Dynamics (MD) Simulations

The initial coordinates of the protein–ligand complexes were obtained from molecular docking. MD simulations were performed using the *Groningen Machine for Chemical Simulations (GROMACS)* [40] software with the CHARMM36 force field [41] for the protein and the CHARMM general force field (CGenFF) for the ligand [42]. The protein–ligand complex was placed in a dodecahedron simulation box with periodic boundary conditions and filled with TIP3P water molecules. Na^+^ and Cl^−^ ions were added to neutralize the system. The system was parametrized, and the geometry was minimized through 5000 steps of the steepest descent method followed by 10,000 steps of the conjugate gradient method to remove any undesirable interactions and relax the system. The system was gradually annealed under an NVT ensemble for 50 ps to reach an optimal temperature of 300 K. This was followed by a 1 ns density equilibration under NPT ensemble conditions, maintaining a constant temperature of 300 K and constant pressure of 1.0 atm. A Langevin thermostat with a collision frequency of 2 ps and a Berendsen barostat with a pressure relaxation time of 1 ps were used to maintain these conditions. The MD simulation employed the particle mesh Ewald method to describe long-range electrostatic interactions with a van der Waals cutoff of 1.2 nm. Production dynamics were then carried out for 100 ns to investigate the behavior of the complex. The average protein–ligand pose over the simulation period was determined using the gmx_cluster module in GROMACS. This method clustered the MD simulation trajectories, employing the GROMOS clustering algorithm with a cutoff of 0.15 nm, to identify the most representative pose of the protein–ligand complex throughout the simulation.

### 3.3. Binding Free Energy Calculations

The binding free energy calculations were conducted on the most converged trajectories identified during root–mean–square deviation (RMSD) analysis. The MM-GBSA (Molecular Mechanics Generalized Born Surface Area) method was used for these calculations, utilizing the gmx_MMPBSA tool [43]. The calculations included contributions from van der Waals interactions, electrostatic energy, and the electrostatic and non-polar components of the solvation-free energy, with the electrostatic contribution computed using the Generalized Born equation and the non-polar contribution estimated by an empirical model. The exterior dielectric constant was set to 78.5 for the solvation energy calculations, and the solute dielectric constant (ε_in_) was set to 1. The polar component of the solvation energy (ΔE_polar_) was calculated using the modified Generalized Born model 2 (GB-OBC2) [44], as developed by Onufriev et al. (referred to as igb = 5 in gmx_MMPBSA). The non-polar component of the solvation energy (ΔE_non-polar_) was determined based on the solvent-accessible surface area (SASA), computed using the Linear Combination of Pairwise Overlaps (LCPO) algorithm [45], with the formula ΔE_non-polar_ = 0.0072 * ΔSASA, with a probe radius of 1.4 Å. Water molecules and added ions were removed from the system prior to the MM-GBSA analysis. The binding free energies (ΔG_total_) of the ligands with the target proteins were calculated using the following formula:

ΔG_Total_ = ΔG_gas_ + ΔG_solv_

where, ΔG_gas_ = ΔE_vdw_ + ΔE_ele_

and, ΔG_solv_ = ΔE_polar_ + ΔE_non-polar_

ΔG_Total_ represents the sum of the gas-phase molecular mechanics energy (ΔG_gas_) and the solvation-free energy (ΔG_solv_). ΔG_gas_ is further divided into van der Waals (ΔE_vdw_) and electrostatic (ΔE_ele_) contributions, while ΔG_solv_ includes polar (ΔE_polar_) and non-polar (ΔE_non-polar_) contributions to the solvation energy. The detailed protocol for this method is available in the previous literature [46]. For each complex, the interaction spectrum between the ligand and the protein target on a per-residue basis was calculated using MM/GBSA decomposition analysis supported by the gmx_MMPBSA tool.

### 3.4. QM/MM-GBSA Calculations

QM/MM-GBSA calculations were performed similarly to MM-GBSA, with the distinction that the quantum mechanical (QM) part specifically employed the semiempirical Hamiltonian PM6-DH+ method [47]. The remainder of the system was treated at the molecular mechanics (MM) level using the ff99SB force field [48]. The QM region encompassed a radius of 5 Å around the binding pocket, comprising both the ligand and amino acid residues from the target protein. This approach enabled the quantum mechanical modeling of interactions within the binding site while efficiently describing the surrounding environment with molecular mechanics.

### 3.5. Symmetry-Adapted Perturbation Theory (SAPT) Calculations

The interaction energies between protein targets and the ligand were analyzed using SAPT calculations performed with the *PSI4* quantum chemistry package [49,50,51]. The methodology involved decomposing these energies into electrostatic (E_ele_), exchange (E_ex_), induction (E_ind_), and dispersion (E_disp_) components. The calculations utilized the jun-cc-pVDZ basis set [52] and employed density fitting for improved computational speed. The initial guess for wavefunction optimization was based on the superposition of atomic densities (SAD), while frozen core approximation was implemented to treat inner electron shells as inert during calculations. To enhance computational efficiency, residues involved in interactions with the ligand were partitioned into smaller blocks, effectively reducing the complexity of SAPT calculations [53]. Each partitioned system underwent separate SAPT analysis, with hydrogen atoms capped to preserve chemical valency and ensure accuracy in quantum mechanical calculations. The interaction energies between each ligand and the protein residues were subsequently obtained by summing the energies calculated from these partitioned systems. We used zeroth-order symmetry-adapted perturbation theory (SAPT0) to balance reasonable accuracy with modest computational cost. This methodological approach facilitated a comprehensive assessment of protein–ligand interactions, offering insights into the specific molecular forces that govern binding affinity.

### 3.6. Conceptual DFT Parameters

We employed Conceptual Density Functional Theory (DFT) to determine the global reactivity parameters of molecular systems using *Gaussian 09* software [54]. The parameters calculated include ionization potential, electron affinity, energy gap, electronegativity, chemical potential, global hardness, global softness, electrophilicity index, nucleophilicity index, and maximum charge transfer index [55,56]. The electronic structure calculations were performed with optimized geometries obtained at the M06-2X/6-311 + G(d,p) level of theory [57].

Molecular interactions and structures were visualized using the *Biovia Discovery Studio visualizer* [38]. Graphs were created with OriginPro 8.5, OriginLab Corporation, Northampton, MA, USA, and GraphPad Prism 5.0a, Boston, MA, USA, www.graphpad.com, while figures were designed with BioRender.com.

## 4. Experimental Details

### 4.1. Chemical Samples

Chemical samples of the compounds were received in powder form. The synthesis and characterization of these compounds have been previously reported [58,59,60,61,62,63]. The stock solutions were prepared by dissolving the compounds in DMSO to a final concentration of 25 mM. Further dilutions were performed manually using DMSO or according to the specific assay protocols.

### 4.2. In Vitro ATX Enzyme Inhibition Assay

Human recombinant ATX (hATX) was purified as previously described [64]. The enzymatic activity of ATX was assessed by measuring the hydrolysis of the synthetic lipid-like FRET-based substrate FS-3 from Echelon Biosciences [64]. Compounds predicted to have potential inhibitory effects through molecular docking were initially screened at a concentration of 10 μM. The reaction mixtures were prepared in 60 μL volumes and loaded into triplicate wells of black-wall 96-well plates. The assay buffer consisted of 50 mM Tris, 140 mM NaCl, 5 mM KCl, 1 mM CaCl_2_, 1 mM MgCl_2_, and 10 μM BSA, with the pH adjusted to 8.0. Each assay contained 4 nM ATX and 1 μM FS-3. The test compounds were diluted in an assay buffer and tested in technical triplicates. For dose–response and IC_50_ determinations, reaction mixtures included 1 μM FS-3, 0 or 4 nM ATX, and varying concentrations of the test compound ranging from 0 to 30 μM. Fluorescence readings were taken at 2 min intervals over 1 h using excitation and emission wavelengths of 485 and 538 nm. Fluorescence data were collected as relative fluorescence units (RFUs) and averaged for the triplicate samples. A compound was considered an experimentally validated hit if it caused more than 50% inhibition of FS-3 hydrolysis at 10 µM concentration. For dose–response studies, nonlinear regression analysis was performed using GraphPad Prism (GraphPad Software, version 10.0.2 (232), San Diego, CA, USA). A variable four-parameter slope model was applied to fit the data, allowing for determining the Hill slope and interpolation of the IC_50_ ± SD. Fluorescence measurements were conducted using the CLARIOstar Plus plate reader from BMG Labtech (BMG Labtech, Ortenberg, Baden-Württemberg, Germany).

### 4.3. Cell Viability Assay

To further characterize the compound, we assessed its effects on the viability of 4T1 murine breast cancer cells and A375 melanoma cells. The 4T1 cells were cultured in RPMI-1640 medium (with L-glutamine (Corning, Durham, NC, USA)), supplemented with 10% charcoal-stripped fetal bovine serum (FBS) (Gibco, ThermoFisher Scientific, Waltham, MA, USA) and 1% penicillin/streptomycin. The cells were maintained at 37 °C in a humidified atmosphere with 5% CO_2_ and passaged before reaching confluence to ensure they remained in the logarithmic growth phase. Similarly, human A375 melanoma cells were purchased from the American Type Culture Collection (ATCC, Manassas, VA, USA) and were cultured in DMEM medium (Corning) supplemented with 10% charcoal-stripped FBS and 1% penicillin/streptomycin. The cells were maintained at 37 °C in a humidified atmosphere containing 5% CO_2_. To measure the antiproliferative effects of the ATX-1d and PTX alone, 750 4T1 or 1500 A375 cells were plated in triplicate in a 96-well plate with either DMSO or nine doses of each test compound. After 72 h of incubation at 37 °C in a humidified 5% CO_2_ atmosphere, cell viability was measured using the CellTiter-Glo assay (Promega, Fitchburg, WI, USA). Luminescence values were measured using a CLARIOstar plate reader (BMG Labtech, Ortenberg, Baden-Württemberg, Germany), and data for each dose were normalized to averaged DMSO-treatment values.

To determine if the potential inhibitor could enhance the efficacy of PTX, 4T1 or A375 cells were plated in triplicate on a 96-well plate with 3 μM ATX inhibitor or matched DMSO for 24 h. The next day, nine doses of PTX were added directly to the pre-treated cells and allowed to incubate for an additional 48 h at 37 °C in a humidified 5% CO_2_ atmosphere. PTX was also added alone for comparison. Cell viability was measured as described in the single compound assay. All viability data were analyzed and plotted using GraphPad Prism (GraphPad Software, version 10.0.2 (232), San Diego, CA, USA). Nonlinear regression analysis was performed using an (inhibitor) vs. normalized response dose–response inhibition model to determine the GI_50_ concentrations for the ATX inhibitor alone or in combination with PTX.

## 5. Results and Discussion

### 5.1. Molecular Docking

**Figure 3:**
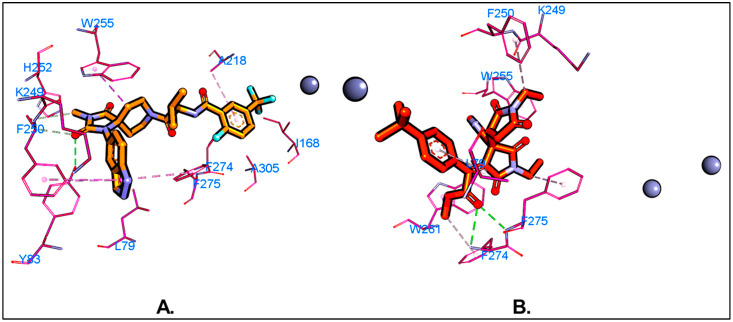
Non-bonding interactions within the binding site residues of ATX (PDB ID: 6W35) for (**A**) native ligand (PDB ID: 6W35) and (**B**) ATX-1d. Zinc ions are depicted using the CPK model. Hydrogen bonds are shown as green dotted lines and halogen bonds as cyan dotted lines, and other non-bonding interactions such as π-π and π-alkyl interactions are represented by pink dotted lines.

We screened a small in-house library of 60 compounds to evaluate their potential to disrupt the ATX-LPA-LPAR signaling axis by targeting ATX (PDB ID: 6W35). Molecular docking, a crucial tool in structure-based drug design, was employed to predict binding affinity between these small molecules and ATX. We validated our docking procedure by docking the native ligand (PDB ID: 6W35) into its binding site, achieving an RMSD of 0.328 between the docking pose and the crystal pose using the *DockRMSD* server [39], which confirms the reliability of our docking protocol. The superimposition of the crystal structure and the best docking pose obtained from PLANTS docking [37] is shown in the Supplementary Information, Figure S1. The native ligand was identified as a Type IV hybrid tunnel–pocket [27] inhibitor at the ATX binding site. Docking analysis, conducted with the *PLANTS* software (version 1.2) [37], assessed various ligand poses for their non-bonding interactions within the ATX binding pocket. Many ligands bound similarly to the native ligand (PDB ID: 6W35), interacting with the crucial hydrophobic pocket and tunnel residues such as ALA218, LEU214, PHE274, PHE211, TRP261, TRP255, PHE275, and LEU79. Compounds with docking scores of −90 or below, calculated using the ChemPLP scoring function [37], were considered hits for the in vitro single-dose ATX enzyme inhibition assay. Given the small size of our library, the threshold was set leniently. Of the 60 compounds, 45 met this criterion (Supplementary Information, Table S1). However, despite not being among the top predicted compounds by docking, ATX-1d was the only compound to exhibit more than 50% inhibition in the enzyme inhibition assay. This discrepancy likely stems from the inherent limitations of molecular docking in accurately estimating binding affinities [65]. Detailed analysis revealed that ATX-1d forms bifurcated hydrogen bonds with PHE274 and PHE275 via its carboxylate group (Figure 3). The pyridine group of ATX-1d positioned itself along the tunnel side, while one N-ethyl succinamide group extended towards the side tunnel region and the other towards the hydrophobic pocket. Recognizing the limitations of molecular docking, we further explored the binding mode and affinity of ATX-1d through MD simulations.

### 5.2. In Vitro Screening through ATX Enzyme Inhibition Assay

**Figure 4:**
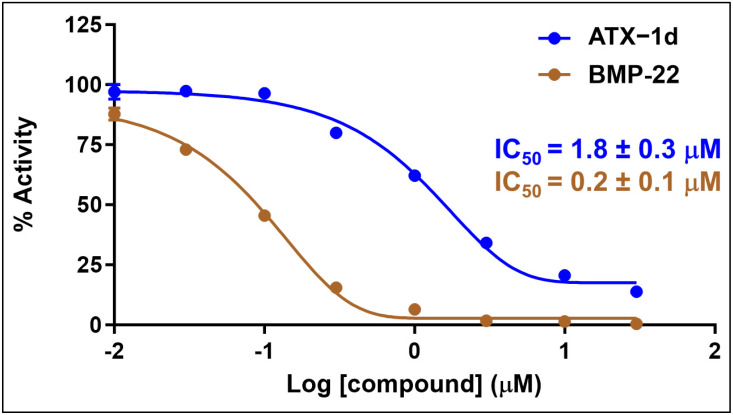
Representative dose–response curve of the ATX enzyme inhibition assay. The hit compound, ATX-1d (blue), and BMP-22 (brown), a previously reported ATX inhibitor, were evaluated for their inhibitory effects on ATX activity. The assay was conducted in duplicate (n = 2), yielding an average IC_50_ of 1.8 ± 0.3 μM and 0.2 ± 0.1 μM for ATX-1d and BMP-22, respectively.

Inhibition assays targeting the ATX-mediated hydrolysis of FS-3 were performed with 45 selected compounds, each at a fixed concentration of 10 μM. Compounds were classified as experimentally validated hits if they achieved more than 50% inhibition of the hydrolysis of the model lysophospholipase D substrate FS-3. From this initial screening, only one compound, ATX-1d, met this criterion (Supplementary Information, Table S1). ATX-1d was then subjected to a dose–response assay to determine its IC_50_ value (Figure 4). This assay, conducted in biological duplicates, produced an average IC_50_ of 1.8 ± 0.3 μM. BMP-22, a previously validated inhibitor of ATX, was used as a control. The second replicate is shown in the Supplementary Information, Figure S2.

### 5.3. MD Simulations

MD simulations provide comprehensive insights into how a drug interacts with its biological target, offering a holistic understanding crucial for guiding the drug discovery process. This approach enables informed decisions regarding lead optimization and rational drug design strategies. Unlike molecular docking, which predicts the spatial orientation of a ligand within the active pocket of a protein, MD simulations account for the dynamic nature of proteins under physiological conditions, considering factors such as conformational stability and flexibility. Our study employed MD simulations to examine the equilibration of binding dynamics of ATX-1d over time. In addition to the widely used MM-GBSA end-point binding free energy calculations, we further employed hybrid QM/MM-GBSA calculations to assess the binding affinity of ATX-1d against the target. The QM/MM-GBSA approach integrates quantum mechanics for the crucial amino acids and the ligand with molecular mechanics for the remainder of the system, potentially offering improved accuracy over MM-GBSA. The enhanced performance of QM/MM-GBSA in predicting binding affinities has been documented in several studies. Mishra and Koca (2018) [66], Wichapong et al. (2014) [67], Pu et al. (2017) [68], and Su et al. (2015) have demonstrated the accuracy of the method when evaluating ligand–target interactions. Our utilization of QM/MM-GBSA thus aims to leverage these benefits for a more reliable prediction of the binding affinity of ATX-1d.

**Figure 5:**
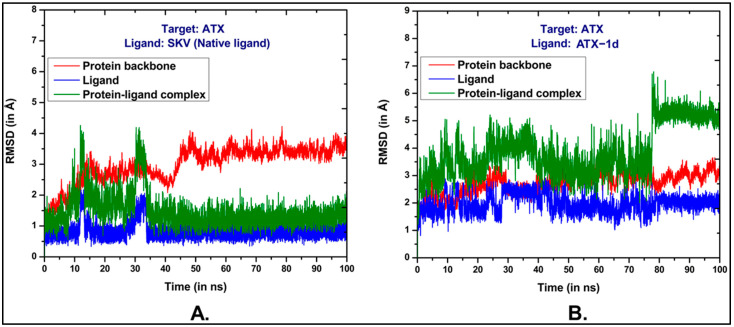
RMSDs obtained through 100 ns MD simulations for (**A**) native ligand (PDB ID: 6W35) and (**B**) ATX-1d against ATX.

**Figure 6:**
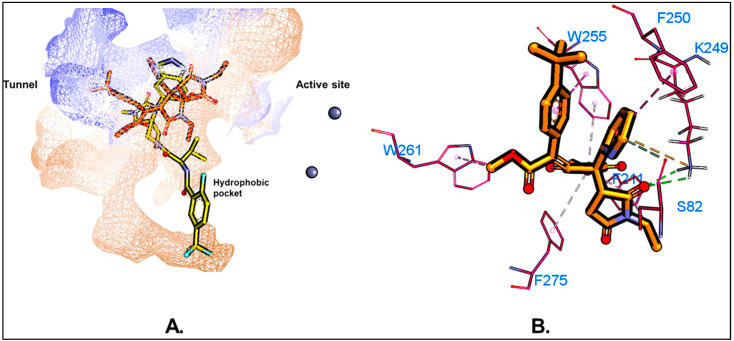
(**A**) Superimposition of the average poses for the native ligand (PDB ID: 6W35) (yellow stick) and ATX-1d (orange stick). The wire mesh represents the protein surface based on hydrophobicity. Blue color represents the hydrophilic regions while brown color represents the hydrophobic regions. (**B**) Non-bonding interactions of ATX-1d in its average pose obtained through MD simulation against ATX. Hydrogen bonds are shown as green dotted lines, halogen bonds as cyan dotted lines, and other non-bonding interactions such as π-π and π-alkyl interactions are represented by pink dotted lines.

**Figure 7:**
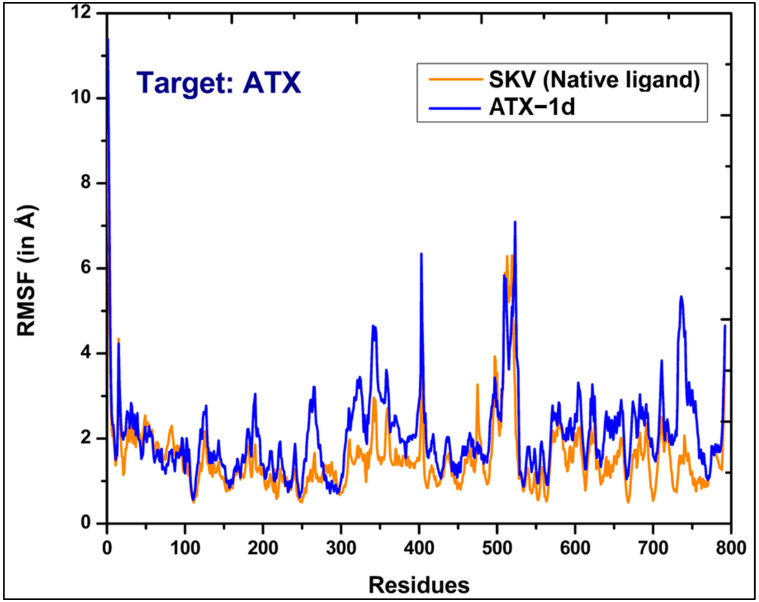
Root–mean–square fluctuation (RMSF) of each residue from ATX in complex with SKV (native ligand) (orange) and ATX-1d (blue).

**Figure 8:**
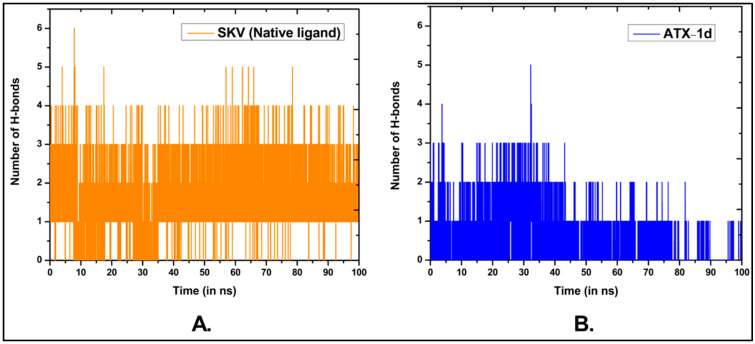
Number of hydrogen bonds determined between the protein and the ligand for (**A**) SKV (native ligand) and (**B**) ATX-1d throughout the 100 ns MD simulations.

**Figure 9:**
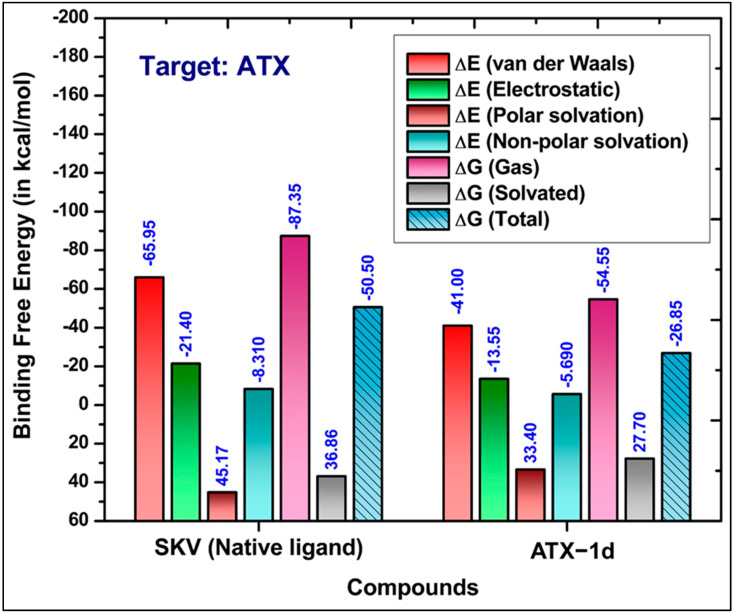
Binding free energies for native ligand (PDB ID: 6W35) and ATX-1d in complex with ATX obtained through the MM-GBSA method [43].

We conducted a 100 ns MD simulation of our hit compound, ATX-1d, in a complex with ATX. The average root–mean–square deviations (RMSDs) were observed to be 2.71 Å for the protein backbone, 1.98 Å for the ligand, and 3.87 Å for the protein–ligand complex (Figure 5B). In comparison, the native ligand (PDB ID: 6W35) exhibited RMSDs of 2.99 Å, 0.86 Å, and 1.48 Å for the protein backbone, ligand, and protein–ligand complex, respectively (Figure 5A). The higher RMSD for the ATX-1d protein–ligand complex suggests some movement of ATX-1d at the binding site compared to its initial docking pose. Nonetheless, ATX-1d remained stably bound at the tripartite binding site of ATX throughout the simulation. Analysis of the average pose over the 100 ns simulation revealed that the native ligand (PBD ID: 6W35) forms hydrogen bonds with tunnel residues TRP255 and SER82 and a halogen bond with the hydrophobic pocket residue PHE274. The residues such as LEU214, PHE250, and TRP261 are also involved in π-π and hydrophobic interactions. Superimposition of the average poses from the MD simulations indicated that ATX-1d does not penetrate deeply into the hydrophobic pocket as the native ligand (PBD ID: 6W35) does (Figure 6A). Instead, it remains positioned between the hydrophobic pocket and the tunnel. For ATX-1d, the average pose showed a bifurcated hydrogen bond with tunnel residue LYS249 and a carbon–hydrogen bond with SER82. The interaction profile also included π-π and hydrophobic interactions with residues TRP255, PHE250, TRP261, PHE275, and PHE211 (Figure 6B). To gain deeper insights into the binding affinity of ATX-1d at the ATX binding site, we extended the production MD simulation by an additional 100 ns, resulting in a cumulative simulation time of 200 ns for the ATX-1d–ATX complex. The RMSD plots for the entire 200 ns simulation of ATX-1d are provided in the Supplementary Information, Figure S3. Analysis of the MD simulation trajectories demonstrated that ATX-1d consistently remained within the binding site of protein for the entire 200 ns of the simulation. There were no occurrences of the ligand dissociating from the binding pocket, suggesting that ATX-1d forms a stable and persistent interaction with the target protein. Figure 7 presents the root–mean–square fluctuation (RMSF) analysis, which measures the deviation of Cα atoms from their reference positions over time. Notably, the RMSF values for ATX-1d exhibit minimal variation when compared to the native ligand, suggesting that ATX-1d maintains the structural stability of the protein similarly to the native ligand (Figure 7). Additionally, hydrogen bond analysis indicates that ATX-1d forms between zero and one hydrogen bonds with ATX, whereas the native ligand forms between one and two hydrogen bonds (Figure 8A,B). Binding free energy calculations on the last 55 ns using the MM-GBSA method [43] yielded a value of −26.85 kcal/mol for ATX-1d, predominantly driven by van der Waals interactions (Figure 9). In contrast, the native ligand (PBD ID: 6W35) exhibited a more stable binding free energy of −50.49 kcal/mol (Figure 9). This indicates that ATX-1d has a lower binding affinity than the native ligand (PDB ID: 6W35). The binding free energy of ATX-1d, calculated over the final 100 ns of the 200 ns simulation, is presented in the Supplementary Information, Table S2. Per residue decomposition of the binding free energy for ATX-1d identified PHE275, TRP255, LYS249, PHE250, PHE211, TRP261, and LEU79 as significant contributors (Table 1). The electrostatic contribution from LYS249 was the highest at −5.32 kcal/mol, reflecting its role in hydrogen bond formation. The residue TRP255 contributed the most through van der Waals interactions at −2.62 kcal/mol, followed by PHE275, located between the pocket and the tunnel. To further validate our findings, we performed QM/MM-GBSA calculations on snapshots from the MD simulation. The binding free energies were −20.02 kcal/mol for the native ligand (PDB ID: 6W35) and −8.37 kcal/mol for ATX-1d (Table 2), consistent with the MM-GBSA results and confirming the moderate binding affinity of ATX-1d towards ATX. The moderate binding affinity of ATX-1d observed in silico was also observed in the in vitro ATX enzyme inhibition assay, which produced the IC_50_ value of 1.8 ± 0.3 μM.

### 5.4. SAPT Calculations

SAPT is a perturbative method known for accurately calculating interaction energies. However, SAPT is computationally intensive, particularly for large systems. We performed a one-step SAPT0 calculation that included all significant residues of the target as one fragment and the ligand as another, which was not feasible due to the substantial computational time required. To address this, we divided the critical residues predicted by the MD simulations of each target into several blocks. We then separately calculated the interaction energy of each block with ATX-1d (Supplementary Information, Table S4). The total interaction energy of ATX-1d with the significant residues was obtained by summing the interaction energies of the individual blocks. This approach relies on the pairwise additivity assumption to estimate the total interaction energy, simplifying the computational process without significantly compromising accuracy. This methodology aligns with the strategy employed by Bamdad et al. (2022) [69] in their study on the decomposition of interaction energies between various flavonoids and Escherichia coli DNA gyrase using the SAPT method. Similarly, Stasyuk et al. applied this approach to calculate interactions in protein–DNA complexes [70]. Recently, Ding et al. performed SAPT calculations to quantify the strength of intermolecular interactions of GPCRs [71]. These previous studies have demonstrated that SAPT can predict interaction energies with an accuracy comparable to the coupled-cluster method, CCSD(T) [72,73,74].

**Figure 10:**
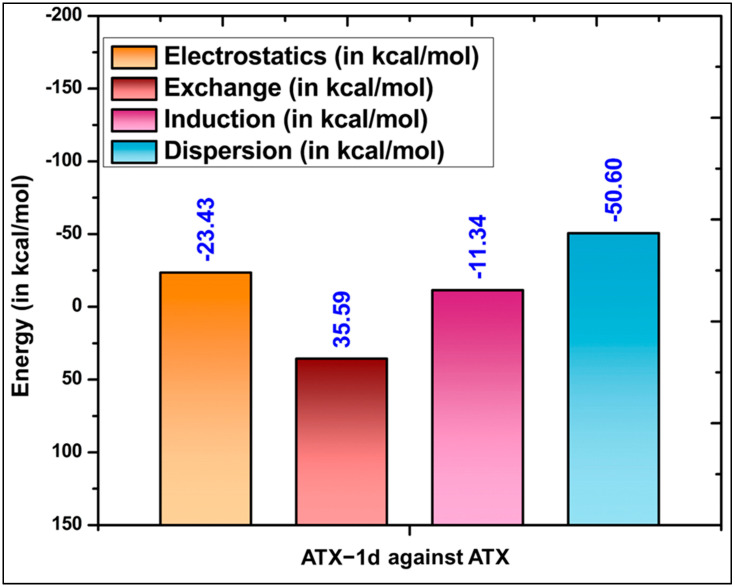
Decomposition of total protein–ligand interaction energy into electrostatic, exchange, induction, and dispersion components [49,50,51].

We evaluated the interactions of ATX-1d with the amino acid residues at the ATX binding site. The analysis revealed that LYS249 exhibited the most stable total SAPT0 interaction energy at −16.83 kcal/mol (Supplementary Information, Table S4), aligning well with the per-residue binding free energy decomposition calculations. The stability is attributed to the bifurcated hydrogen bond formation between ATX-1d and LYS249, resulting in a significant contribution from the electrostatic component. Following LYS249, PHE275 and PHE211 were identified with total SAPT0 interaction energies of −7.74 kcal/mol and −7.60 kcal/mol, respectively. These residues showed the highest contributions of the dispersion component, which can be explained by the presence of π-π and hydrophobic interactions. Based on their total SAPT0 interaction energies, other significant residues included TRP255, LEU244, SER82, and TYR215. The overall interaction energy for ATX-1d was determined to be −49.78 kcal/mol, with the dispersion component contributing the most (Table 3, Figure 10). For comparison, we performed SAPT0 calculations for the native ligand (PDB ID: 6W35), which showed a total interaction energy of −65.80 kcal/mol (Table 3) (Supplementary Information, Table S3). This comparison underscores the strength of the binding affinity of the native ligand (PDB ID: 6W35), reflected in its lower (more negative) interaction energy.

Our comprehensive computational analysis, encompassing molecular docking, MD simulations, MM-GBSA, QM/MM-GBSA, and SAPT0 calculations, indicates that ATX-1d exhibits significant activity against ATX. The pharmacokinetic properties and drug-likeness of ATX-1d were predicted using the *SwissADME* [75] and *pkCSM* web servers [76]. *SwissADME* provided insights into key drug-like properties such as lipophilicity, solubility, and absorption, while *pkCSM* predicted various pharmacokinetic parameters, including ADMET (Absorption, Distribution, Metabolism, Excretion, and Toxicity) profiles. The results from these computational predictions are summarized in the Supplementary Information, Tables S5 and S6. To evaluate the potential off-target effects of ATX-1d, we utilized the *SwissTargetPrediction* webserver [77]. The SMILES code of ATX-1d was submitted to this online tool, which predicts possible biological targets based on chemical similarity to known ligands. The analysis yielded low probability scores for other potential targets, suggesting that ATX-1d likely exhibits minimal off-target effects (Supplementary Information, Table S7). Consequently, the observed biological effects are predominantly attributable to the action of ATX-1d on its primary targets.

### 5.5. Cellular Activity of ATX-1d

To further evaluate the anticancer potential of our hit compound, ATX-1d, we investigated its cellular activity against 4T1 murine breast cancer cells and A375 human melanoma cells using cell viability assays. As expected, each cell line shows a potent response to PTX treatment over three days (Table 4). In contrast, treatment with ATX-1d as a single agent does not reduce cell viability below 50% in either cell line, even at the highest dose, 20 μM. These data indicate that ATX-1d treatment alone is ineffective at impairing cell viability, and aligns with previous findings that ATX inhibitors are non-cytotoxic [78,79].

### 5.6. ATX Inhibition by ATX-1d Improves Cellular Response to PTX

**Figure 11:**
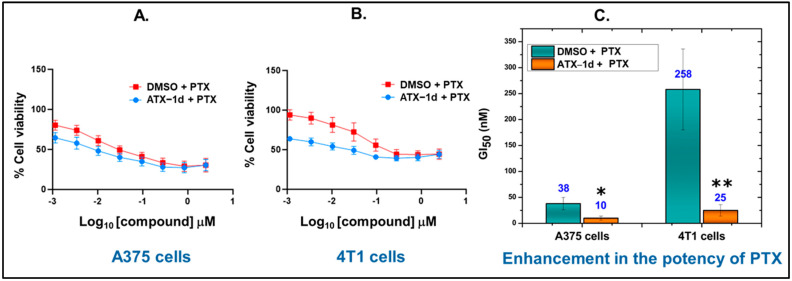
Synergistic effects of ATX-1d with PTX in multiple cancer cell lines. (**A**) A375 cells were treated for 24 h with 3 μM ATX-1d or DMSO before increasing concentrations of PTX were added for an additional 48 h. Cell viability was determined using CellTiter-Glo and all values were normalized to DMSO-treated samples to obtain the final percent cell viability. The red line indicates the percent cell viability obtained at each dose with paclitaxel treatment in combination with DMSO, and the blue line indicates the same for paclitaxel in combination with ATX-1d (n = 3 biological replicates). (**B**) Cell viability data for 4T1 cells performed as in (**A**) (n = 3 biological replicates). (**C**) Bar graph displays the enhancement in PTX potency by showing extracted GI_50_ values for each replicate and condition. The error bars represent the standard deviation (SD) obtained from biological triplicates. The GraphPad Prism was used to conduct two-tailed Student’s *t*-tests for analyzing independent groups in the synergistic assay. Significance levels are denoted as follows: * *p* < 0.05, ** *p* < 0.01.

Given the role of ATX and LPA in pro-survival signaling, we next investigated whether ATX inhibition by ATX-1d could be used in combination with PTX to enhance the cellular response to PTX treatment [80]. To determine this, A375 cells were exposed to increasing concentrations of PTX for 48 h, with or without a 24 h pretreatment with 3 μM ATX-1d, a concentration that does not have any observable cytotoxicity by itself (Table 4). The combination of PTX and ATX-1d resulted in a fourfold increase in the potency of PTX, with the GI_50_ improving from 38 ± 12 nM (PTX alone) to 10 ± 0 nM (combination, Figure 11A,C). Remarkably, in 4T1 cells, ATX-1d also increases the potency of PTX tenfold, reducing the GI_50_ from 258 ± 78 nM (PTX alone) to 25 ± 11 nM (combination treatment, Figure 11B,C).

### 5.7. Conceptual DFT Parameters

The global reactive parameters of ATX-1d, displayed in Table 5, provide critical insights into its chemical behavior. By examining frontier molecular orbitals (FMOs), specifically the highest occupied (HOMO) and lowest unoccupied (LUMO) orbitals, chemists can understand the reactivity and regioselectivity of ATX-1d. Important descriptors such as ionization potential (IP), electron affinity (EA), HOMO-LUMO energy gap (E_g_), electronegativity (χ), chemical potential (μ), global hardness (η), global softness (S), electrophilicity index (ω), nucleophilicity index (ε), and maximum charge transfer index (ΔN_max_) were calculated.

## 6. Conclusions

Cancer therapeutic resistance remains a significant obstacle in oncology, compromising treatment efficacy and patient outcomes. Recent studies indicate that the inhibition of ATX can significantly enhance the efficacy of chemotherapeutic agents and prevent the development of resistance, providing a potential pathway for more effective cancer treatments. This study utilized computational methods alongside in vitro assays to identify and evaluate ATX-1d, a novel compound targeting ATX. ATX-1d demonstrated an IC_50_ of 1.8 (±0.3) μM in the ATX enzyme inhibition assay. Computational studies revealed that ATX-1d exhibits significant activity against ATX. The binding affinities were evaluated using MM-GBSA, QM/MM-GBSA, and SAPT methods, consistently indicating effective interactions with ATX. Molecular dynamics simulations highlighted that ATX-1d binds stably within the tunnel and hydrophobic regions of the ATX tripartite binding site, with LYS249 forming a critical bifurcated hydrogen bond contributing to the binding stability. Importantly, ATX-1d exhibited no cytotoxicity as a single agent in the cell viability assay, but significantly enhanced the efficacy of paclitaxel, increasing its potency fourfold in A375 melanoma cells and tenfold in 4T1 breast carcinoma cells. These results establish ATX-1d as a promising hit compound for further optimization and in vivo studies. This discovery also validates a novel pharmacophore model for developing next-generation ATX inhibitors. The insights gained from these computational and experimental analyses will guide the refinement of the inhibitory mechanism of ATX-1d and inform the future development of therapeutic strategies.

## Figures and Tables

**Table 1:** Per-residue decomposition of the binding free energy for ATX-1d in complex with ATX, obtained using the MM-GBSA method. The energy values are given in kcal/mol.

Residues	van der Waals		Electrostatic		Polar Solv.		Non-Polar Solv.		TOTAL	
	Avg.	Std. Err. of Mean	Avg.	Std. Err. of Mean	Avg.	Std. Err. of Mean	Avg.	Std. Err. of Mean	Avg.	Std. Err. of Mean
GLN:67	−0.09	0.00	−0.46	0.00	0.45	0.00	−0.04	0.00	−0.14	0.00
LEU:79	−1.29	0.01	0.06	0.00	−0.21	0.00	−1.22	0.00	−2.66	0.01
SER:82	−0.47	0.00	−0.06	0.00	−0.08	0.00	−0.43	0.00	−1.03	0.01
TYR:83	−0.78	0.01	−0.55	0.01	0.13	0.00	−0.65	0.01	−1.85	0.02
PHE:211	−1.52	0.01	−0.39	0.00	0.11	0.00	−1.33	0.01	−3.13	0.01
TYR:215	−0.93	0.01	−0.20	0.00	0.02	0.00	−0.72	0.00	−1.83	0.01
LEU:244	−0.74	0.00	0.21	0.00	−0.26	0.00	−0.70	0.00	−1.48	0.01
ARG:245	−0.09	0.00	0.20	0.00	−0.20	0.00	0.00	0.00	−0.10	0.01
LYS:249	−1.83	0.00	−5.32	0.03	4.15	0.02	−1.54	0.00	−4.53	0.03
PHE:250	−1.78	0.00	−0.27	0.00	0.17	0.00	−1.45	0.00	−3.34	0.01
TRP:255	−2.63	0.01	−0.60	0.00	−0.13	0.00	−1.92	0.01	−5.28	0.01
TRP:261	−1.43	0.01	−0.10	0.00	−0.09	0.00	−1.25	0.00	−2.86	0.01
ILE:262	−0.26	0.00	0.00	0.00	0.01	0.00	−0.22	0.00	−0.46	0.00
THR:273	−0.18	0.00	−0.02	0.00	0.10	0.00	−0.15	0.00	−0.25	0.00
PHE:274	−0.41	0.00	−0.08	0.00	0.06	0.00	−0.18	0.00	−0.60	0.00
PHE:275	−2.44	0.01	−0.66	0.00	0.06	0.00	−2.02	0.00	−5.06	0.01

**Table 2:** Results of QM/MM-GBSA calculations for the ligand and amino acid residues within the 5 Å radius of the ligand. The energy values are given in kcal/mol.

Compounds	van der Waals		Electrostatics (SCF)		Polar Solv.		Non-Polar Solv.		TOTAL	
	Avg.	Std. Err. of Mean	Avg.	Std. Err. of Mean	Avg.	Std. Err. of Mean	Avg.	Std. Err. of Mean	Avg.	Std. Err. of Mean
SKV (native ligand, PDB ID: 6W35) vs. ATX	−5.85	0.01	−68.09	0.11	58.78	0.11	−7.79	0.01	−22.95	0.06
ATX−1d vs. ATX	−7.06	0.06	−65.58	0.43	69.86	0.46	−5.6	0.02	−8.37	0.11

**Table 3:** Results of SAPT0 calculations for the ligand and interacting amino acid residues. The energy values are given in kcal/mol.

Compounds	Total SAPT0 Protein–Ligand Interaction Energy (in kcal/mol)
SKV (native ligand, PDB ID: 6W35) vs. ATX	−65.80
ATX-1d vs. ATX	−49.78

**Table 4:** Cell growth assay for cytotoxicity. Indicated cell lines were plated in triplicate with nine serial dilutions of either ATX-1d or PTX for three days, and cell viability was determined using the CellTiter-Glo assay. Calculated GI_50_ values are shown below for each agent with the standard error of the mean. The “>20” indicates that the highest dose used in the assay did not reduce cell viability below 50% (n = 3 biological replicates for ATX-d treatment and two biological replicates for PTX treatment).

4T1 Breast Cancer Cells		A375 Melanoma Cells	
Compounds	GI_50_ (in nM)	Compounds	GI_50_ (in nM)
Paclitaxel	62 ± 18	Paclitaxel	4 ± 1
ATX-1d	>20,000	ATX-1d	>20,000

**Table 5:** Conceptual DFT parameters calculated at M06-2X/6-311 + G(d,p) level of theory.

Compound	ATX−1d
E_LUMO_	−0.041
E_HOMO_	−0.299
Energy Gap (E_g_) {E_g_ = E_LUMO_ − E_HOMO_}	0.258
Ionization Potential (IP) {IP = −E_HOMO_}	0.299
Electron Affinity (EA) {EA = −E_LUMO_}	0.041
Electronegativity (χ) {χ = $\frac{-(E_{\mathrm{LUMO}}+E_{\mathrm{HOMO}})}{2}\}$	0.170
Chemical Potential (μ) {μ = $\frac{\left(E_{\mathrm{LUMO}}+E_{\mathrm{HOMO}}\right)}{2}\}$	−0.170
Global Hardness (η) {η = $\frac{E_{g}}{2}$}	0.129
Global Softness (S) {S = $\frac{1}{2\eta }$}	3.875
Electrophilicity Index (ω) {ω = $\frac{\mu^{2}}{2\eta }$}	0.112
Nucleophilicity Index (ε) {ε = $\frac{1}{\omega }$}	8.964
Maximum Charge Transfer Index (ΔN_max_) {ΔN_max_ = $\frac{-\mu }{\eta }$}	1.315

## Data Availability

The data supporting this article have been included in the main text or as part of the Supplementary Information. The computational chemistry files, including molecular docking output files, molecular dynamics simulation trajectories and output files, SAPT files, and conceptual DFT files, can be made available on request.

## Supplementary Materials

The following supporting information can be downloaded at: https://www.mdpi.com/article/10.3390/molecules29184285/s1, Figure S1: Superimposition of the crystal pose (yellow) and the docking pose (pink) demonstrates the accuracy of the docking procedure. The evaluation was conducted to validate this process, confirming that the docked pose of the native ligand aligns well with its crystal structure. The root–mean–square deviation (RMSD) between the crystal and docking poses was calculated to be 0.328 Å using the DockRMSD webserver, as detailed in the main text. Table S1: SMILES notation, docking scores, and % inhibition of 60 compounds tested at 10 μM concentration. Figure S2: A second replicate of the in vitro autotaxin enzyme inhibition assay was conducted. The average IC_50_ values obtained from the biological duplicates were 1.8 ± 0.3 μM for ATX-1d and 0.2 ± 0.1 μM for BMP-22, as mentioned in the main text. Figure S3: RMSD plot for the ATX-1d–ATX complex obtained through the 200 ns MD simulation. Table S2: Binding free energy of ATX-1d calculated using the MM-GBSA method for the last 100 ns of the 200 ns MD simulation. Table S3: Results of the SAPT0 calculations for the SKV (native ligand, PDB ID: 6W35)–ATX complex. Table S4: Results of the SAPT0 calculations for the ATX-1d-ATX complex. Table S5: Evaluation of pharmacokinetics, drug-likeness, and medicinal chemistry friendliness predictions of ATX-1d through *SwissADME* webserver. Table S6: Prediction of ADMET properties of ATX-1d through *pkCSM* webserver based on graph-based signatures. Table S7: Results of the *SwissTargetPrediction* webserver.

## Abbreviations

ATX, autotaxin; LPAR, lysophosphatidic acid receptor; LPA, lysophosphatidic acid; GPCR, G protein-coupled receptor; TME, tumor microenvironment; PI3K, phosphoinositide 3-kinase; AKT, protein kinase B; RAS, rat sarcoma; MAPK, mitogen-activated protein kinase; DNA, deoxyribonucleic acid; NF-κB, nuclear factor kappa B; NRF2, nuclear factor erythroid 2-related factor 2; HDAC, histone deacetylase; EZH2, enhancer of zeste homolog 2; FDA, food and drug administration; MMFF94, Merck Molecular Force Field 1994; MM-GBSA, molecular mechanics/generalized Born surface area; QM/MM-GBSA, quantum mechanics/molecular mechanics/generalized Born surface area; SAPT, symmetry-adapted perturbation theory; DFT, density functional theory; PTX, paclitaxel; PDB, protein databank; RMSD, root–mean–square deviation; GROMOS96, GROningen MOlecular Simulation 1996; GROMACS, GROningen MAchine for Chemical Simulations; CHARMM36, Chemistry at HARvard Macromolecular Mechanics (version 36); MD, molecular dynamics; TIP3P, Three Point Charge Model (TIP3P); NVT, number of particles (N)–volume (V)–temperature (T); NPT, number of particles–pressure–temperature; SASA, solvent-accessible surface area; LCPO, Linear Combination of Pairwise Overlaps; PM6-DH+, Parametric Method 6 with Dispersion and Hydrogen bonding corrections; ff99SB, Force Field 1999 Side-Chain and Backbone; QM, quantum mechanics; MM, molecular mechanical; SAD, superposition of atomic densities; hATX, human recombinant ATX; FBS, fetal bovine serum; CCSD(T), Coupled Cluster with Single and Double Excitations and Perturbative Triple Excitations.
