# Supplementary material for: Novel Autotaxin Inhibitor ATX-1d Significantly Enhances Potency of Paclitaxel—An In Silico and In Vitro Study
Source: Molecules. 2024 Sep 10;29(18):4285. doi: 10.3390/molecules29184285 (PMC11434342; doi:10.3390/molecules29184285)
Supplement: Supplementary file 1 [file molecules-29-04285-s001.zip › molecules-3171128-supplementary.pdf]

Novel Autotaxin Inhibitor ATX-1d Significantly Enhances Potency of Paclitaxel- An *In Silico* and *In Vitro* Study

Prateek Rai[1,2#], Christopher J. Clark[1,2#], Carl B. Womack[3], Curtis Dearing[2], Joshua Thammathong[2], Derek D. Norman[4], Gabor J. Tigyi[4], Subhabrata Sen[5], Kevin Bicker[1,2], April M. Weissmiller[1,3], Souvik Banerjee*[1,2]

#: These authors have equal contributions. *: Corresponding author.

[1]Molecular Biosciences, Middle Tennessee State University, Murfreesboro, TN, USA. [2]Department of Chemistry, Middle Tennessee State University, Murfreesboro, TN, USA. [3]Department of Biology, Middle Tennessee State University, Murfreesboro, TN, USA. [4]Department of Physiology, University of Tennessee Health Science Center, Memphis, TN, USA. [5]Department of Chemistry, Shiv Nadar Institution of Eminence Deemed to be University, Dadri, Chithera, Gautam Buddha Nagar, India.

Figure S1: Superimposition of the crystal pose (yellow) and the docking pose (pink) demonstrates the accuracy of the docking procedure. The evaluation was conducted to validate this process, confirming that the docked pose of the native ligand aligns well with its crystal structure. The root mean square deviation (RMSD) between the crystal and docking poses was calculated to be 0.328 Å using the DockRMSD webserver, as detailed in the main text.

Table S1: SMILES notation, docking scores, and % inhibition of 60 compounds tested at 10 μM concentration.

Figure S2: A second replicate of the in vitro autotaxin enzyme inhibition assay was conducted. The average IC_{50} values obtained from the biological duplicates were 1.8 ± 0.3 μM for ATX-1d and 0.2 ± 0.1 μM for BMP-22, as mentioned in the main text.

Figure S3: RMSD plot for the ATX-1d-ATX complex obtained through the 200 ns MD simulation.

Table S2: Binding free energy of ATX-1d calculated using the MM-GBSA method for the last 100 ns of the 200 ns MD simulation.

Table S3: Results of the SAPT0 calculations for the SKV (native ligand, PDB ID: 6W35)-ATX complex.

Table S4: Results of the SAPT0 calculations for the SKV (native ligand, PDB ID: 6W35)-ATX complex.

Table S5: Evaluation of pharmacokinetics, drug-likeness, and medicinal chemistry friendliness predictions of ATX-1d through *SwissADME* webserver.

Table S6: Prediction of ADMET properties of ATX-1d through *pkCSM* webserver based on graph-based signatures.

Table S7: Results of the *SwissTargetPrediction* webserver.

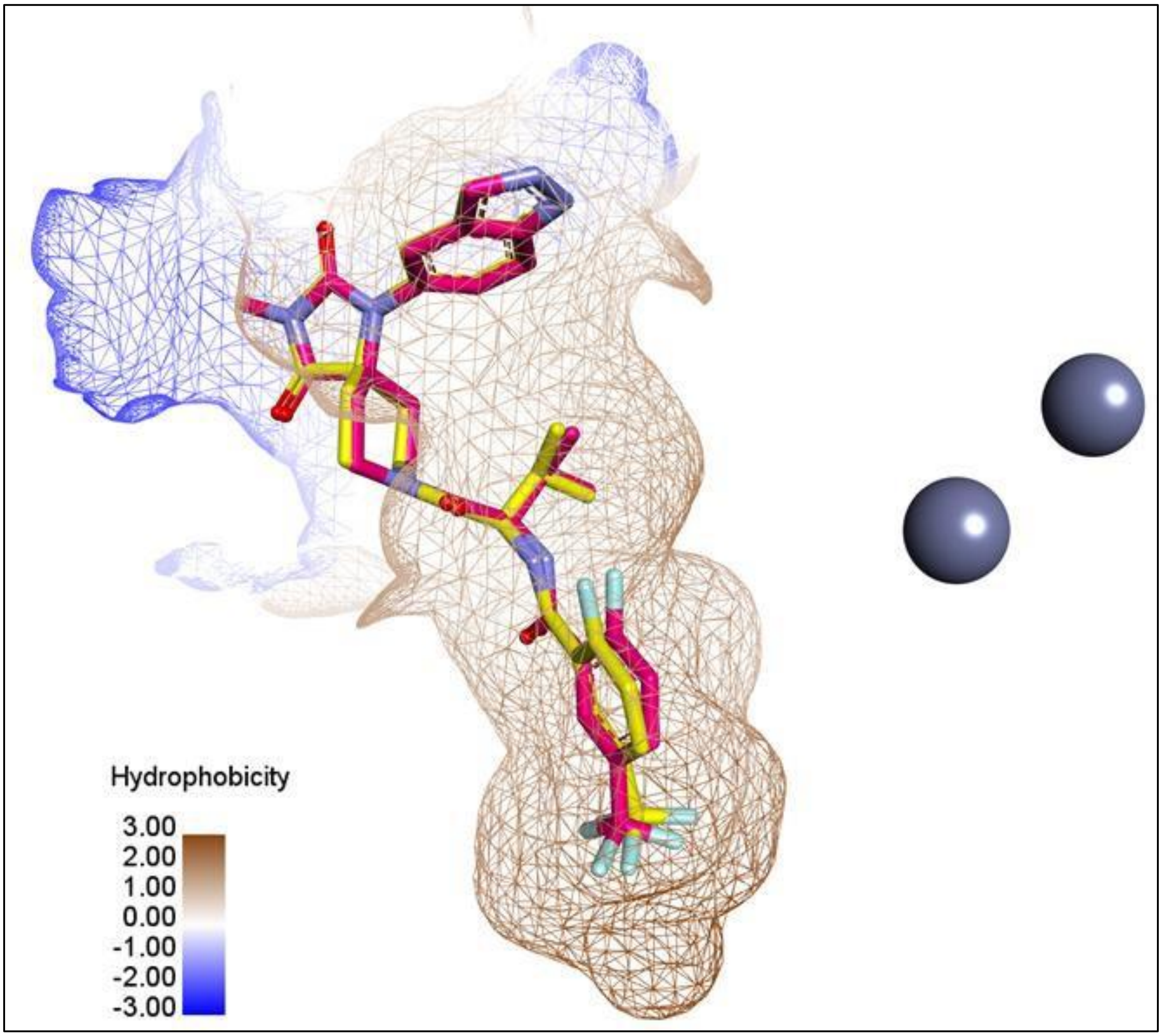

Figure S1: Superimposition of the crystal pose (yellow stick) and the docking pose (pink stick) demonstrates the accuracy of the docking procedure. Zinc ions are depicted using the CPK model. A control study was conducted to validate this process, confirming that the docked pose of the native ligand aligns well with its crystal structure. The root-mean-square deviation (RMSD) between the crystal and docking poses was calculated to be 0.328 Å using the *DockRMSD* webserver, as detailed in the main text.

Table S1: SMILES notation, docking scores, and % inhibition of 60 compounds tested at 10 μM concentration.

Compound ID	SMILES	PLANTS docking score	% inhibition in ATX activity assay at 10 μM
ATX-1a	O=C1N(C)C(/C(C1(C2=NC=CC=C2)C(C(N3C)=O)CC3=O)=C(C(OC)=O)/C4=CC=C(C(C)(C)C)C=C4)=O	-110.57	42.50
ATX-1b	O=C1N(CC)C(/C(C1(C2=NC=CC=C2)C(C(N3CC)=O)CC3=O)=C(C(OC)=O)/C4=CC=CC=C4)=O	-94.97	3.59
ATX-1c	O=C1N(C)C(/C(C1(C2=NC=CC=C2)C(C(N3C)=O)CC3=O)=C(C(OC)=O)/C4=CC=CC=C4)=O	-88.07	Not tested
**ATX-1d**	**O=C1N(CC)C(/C(C1(C2=NC=CC=C2)C(C(N3CC)=O)CC3=O)=C(C(OC)=O)/C4=CC=C(C(C)(C)C)C=C4)=O**	**-91.73**	**82.91**
ATX-1e	O=C1N(C)C(/C(C1(C2=NC=CC=C2)C(C(N3C)=O)CC3=O)=C(C(OC)=O)/C4=CC=C(F)C=C4)=O	-87.40	Not tested
ATX-1f	O=C1N(C)C(/C(C1(C2=NC=CC=C2)C(C(N3C)=O)CC3=O)=C(C(OCC)=O)/C4=CC=C(C)C=C4)=O	-91.48	-0.36
ATX-1g	O=C1N(C)C(/C(C1(C2=NC=CC=C2)C(C(N3C)=O)CC3=O)=C(C(OC)=O)/C4=CC=C(Cl)C=C4)=O	-83.65	Not tested
ATX-1h	O=C1N(CC)C(/C(C1(C2=NC=CC=C2)C(C(N3CC)=O)CC3=O)=C(C(OC)=O)/C4=CC=CC(OC)=C4)=O	-98.82	7.60
ATX-1i	O=C1N(CC)C(/C(C1(C2=NC=CC=C2)C(C(N3CC)=O)CC3=O)=C(C(OC)=O)/C4=CC=C(F)C=C4)=O	-105.71	10.04
ATX-1j	O=C1N(CC)C(/C(C1(C2=NC=CC=C2)C(C(N3CC)=O)CC3=O)=C(C(OC)=O)/C4=CC=C(Cl)C=C4)=O	-87.60	Not tested
ATX-2a	O=C1C2([H])C(CC)=N[C@](C(OC)=O)(C3=CC=C(Br)C=C3)[C@]2([H])C(N1C)=O	-91.16	18.99
ATX-2b	O=C1C2([H])C(CC)=N[C@](C(OC)=O)(C3=CC=C(Br)C=C3)[C@]2([H])C(N1CC4=CC=CC=C4)=O	-105.36	24.88
ATX-2c	O=C1C2([H])C(CC)=N[C@](C(OC)=O)(C3=CC=CC(OC)=C3)[C@]2([H])C(N1C)=O	-96.79	13.04
ATX-2d	O=C1C2([H])C(C)=N[C@](C(OC)=O)(C3=CC=CC(OC)=C3)[C@]2([H])C(N1CC)=O	-98.71	4.61
ATX-2e	O=C1C2([H])C(CC)=N[C@](C(OC)=O)(C3=CC=C(Br)C=C3)[C@]2([H])C(N1CC)=O	-93.43	10.06
ATX-2f	O=C1C2([H])C(C)=N[C@](C(OC)=O)(C3=CC=C(Br)C=C3)[C@]2([H])C(N1CC)=O	-89.16	Not tested
ATX-2g	O=C1C2([H])C(CC)=N[C@](C(OC)=O)(C3=CC=CC=C3)[C@]2([H])C(N1CC4=CC=CC=C4)=O	-99.27	3.38
ATX-2h	O=C1C2([H])C(C)=N[C@](C(OC)=O)(C3=CC=CC=C3)[C@]2([H])C(N1C)=O	-85.17	Not tested
ATX-2i	O=C1C2([H])C(C)=N[C@](C(OC)=O)(C3=CC=C(Br)C=C3)[C@]2([H])C(N1C)=O	-88.55	Not tested

ATX-2j	O=C1C2([H])C(C)=N[C@](C(OC)=O)(C3=CC=C(Br)C=C3)[C@]2([H])C(N1CC4=CC=CC=C4)=O	-110.48	No effect
ATX-3a	O=C1C(C(C2=CC=CC=C2)OC3(C(OC)=O)C4=CC=CC(OC)=C4)C3C(N1CC5=CC=CC=C5)=O	-115.32	No effect
ATX-3b	O=C(N1CC2=CC=CC=C2)C3C(C(C4=CC=CC=C4)OC3(C(OC)=O)C5=CC=C(C(C)(C)C)C=C5)C1=O	-120.22	33.65
ATX-3c	O=C(N1C2=CC=CC=C2)C3C(C(C4=CC=C(F)C=C4F)OC3(C(OC)=O)C5=CC=C(Cl)C=C5)C1=O	-116.81	-35.86
ATX-3d	O=C(N1CC2=CC=CC=C2)C3C(C(C4=CC(OC)=CC=C4)OC3(C(OC)=O)C5=CC=CC(Cl)=C5)C1=O	-116.59	No effect
ATX-3e	O=C(N1CC2=CC=CC=C2)C3C(C(C4=CC=CC=C4)OC3(C(OC)=O)C5=CC=C(C(C)(C)C)C=C5)C1=O	-120.36	7.82
ATX-3f	O=C(N1C2=CC=CC=C2)C3C(C(C4=CC(OC)=CC=C4)OC3(C(OC)=O)C5=CC=C(Cl)C=C5)C1=O	-110.45	5.80
ATX-3g	O=C(N1C2=CC=CC=C2)C3C(C(C4=CC(OC)=CC=C4)OC3(C(OC)=O)C5=CC=CC=C5)C1=O	-113.32	-30.09
ATX-3h	O=C(N1CC)C2C(C(C3=CC(F)=CC=C3)OC2(C(OC)=O)C4=CC=C(C(C)(C)C)C=C4)C1=O	-113.40	-5.76
ATX-3i	O=C(N1C2=CC=CC=C2)C3C(C(C4=CC(F)=CC=C4)OC3(C(OC)=O)C5=CC=CC=C5)C1=O	-113.74	-37.39
ATX-3j	O=C(N1CC2=CC=CC=C2)C3C(C(C4=CC(Cl)=CC=C4)OC3(C(OC)=O)C5=CC=CC(OC)=C5)C1=O	-119.59	16.18
ATX-4a	CC(CC(OCCCCOC(C1=CC=C(Br)C=C1)C(OC)=O)=C2)(C)CC2=O	-114.29	18.74
ATX-4b	CC(CC(OCCCCOC(C1=CC=CC(OC)=C1)C(OC)=O)=C2)(C)CC2=O	-110.50	12.62
ATX-4c	CC(CC(OCCCCOC(C1=CC=C(C(C)(C)C)C=C1)C(OC)=O)=C2)(C)CC2=O	-116.59	-19.64
ATX-4d	O=C1C=C(OCCCCOC(C2=CC=C(Cl)C(Cl)=C2)C(OC)=O)CC(C)(C)C1	-110.91	14.79
ATX-4e	CC(CC(OCCCCOC(C1=CC=CC=C1C)C(OCC2=CC=CC=C2)=O)=C3)(C)CC3=O	-129.30	33.65
ATX-4f	CC(CC(OCCCCOC(C1=CC=CC(Cl)=C1)C(OC)=O)=C2)(C)CC2=O	-114.95	40.73
ATX-4g	CC(CC(OCCCCOC(C1=CC=CC=C1)C(OC)=O)=C2)(C)CC2=O	-103.61	32.57
ATX-4h	O=C1C=C(OCCCCOC(C2=CC=CC=C2OC)C(OCC3=CC=C(OCO4)C4=C3)=O)CC(C)(C)C1	-136.41	36.05
ATX-4i	CC(CC(OCCCCOC(C1=CC=CC(C(F)(F)F)=C1)C(OC)=O)=C2)(C)CC2=O	-119.72	15.21
ATX-4j	CC(CC(OCCCCOC(C1=CC=CC(F)=C1)C(OC)=O)=C2)(C)CC2=O	-111.78	41.37
ATX-5a	O=C2N([H])C1=CC=CC=C1C32C(C(OC)=O)C3C(OCC)=O	-89.26	Not tested
ATX-5b	O=C2N([H])C1=C(Cl)C=CC=C1C32C(C(OC)=O)C3C(OCC)=O	-91.14	No effect
ATX-5c	O=C2N(C(C)=O)C1=CC=CC=C1C32C(C(OCC)=O)C3C(OCC)=O	-102.04	-4.52

ATX-5d	O=C2N([H])C1=CC=C(F)C=C1C32C(C(OC)=O)C3C(OCC)=O	-91.61	No effect
ATX-5e	O=C2N([H])C1=C(Cl)C=CC=C1C32C(C(OCC)=O)C3C(OCC)=O	-97.98	57.94
ATX-5f	O=C2N([H])C1=CC=C(Br)C=C1C32C(C(OC)=O)C3C(OCC)=O	-92.20	No effect
ATX-5g	O=C2N(C(OC(C)(C)C)=O)C1=CC=C(Br)C=C1C32C(C(OCC)=O)C3C(OCC)=O	-95.30	-0.48
ATX-5h	O=C2N([H])C1=CC=C(OC)C=C1C32C(C(OCC)=O)C3C(OCC)=O	-92.70	No effect
ATX-5i	O=C2N(C(C)=O)C1=CC=C(OC)C=C1C32C(C(OCC)=O)C3C(OCC)=O	-100.65	No effect
ATX-5j	O=C2N(C(C)=O)C1=CC=C(C)C=C1C32C(C(OCC)=O)C3C(OCC)=O	-101.87	-10.18
ATX-6a	O=C([O-])C(C2=CC=C(C)C=C2)[N+]1(C)CCOCC1	-82.72	Not tested
ATX-6b	O=C([O-])C(C2=CC=C(C(C)(C)C)C=C2)[N+]1(C)CCOCC1	-93.34	No effect
ATX-6c	O=C([O-])C(C2=CC=C(Cl)C=C2)[N+]1(C)CCOCC1	-81.99	Not tested
ATX-6d	O=C([O-])C(C2=CC=C(Br)C=C2)[N+]1(C)CCOCC1	-81.73	Not tested
ATX-6e	O=C([O-])C(C2=CC(C(F)(F)F)=CC=C2)[N+]1(C)CCOCC1	-88.09	Not tested
ATX-6f	C[N+]1(C(C([O-])=O)C2=CSC=C2)CCOCC1	-75.26	Not tested
ATX-6g	[O-]C(C(C3=CC(C(F)(F)F)=CC=C3)[N+]1(CC2)CCN2CC1)=O	-93.23	No effect
ATX-6h	[O-]C(C(C3=CC=C(Cl)C=C3)[N+]1(CC2)CCN2CC1)=O	-82.70	Not tested
ATX-6i	[O-]C(C(C3=CC(Cl)=CC=C3)[N+]1(CC2)CCN2CC1)=O	-89.12	Not tested.
ATX-6j	[O-]C(C(C3=CC(OC)=CC=C3)[N+]1(CC2)CCN2CC1)=O	-87.22	Not tested

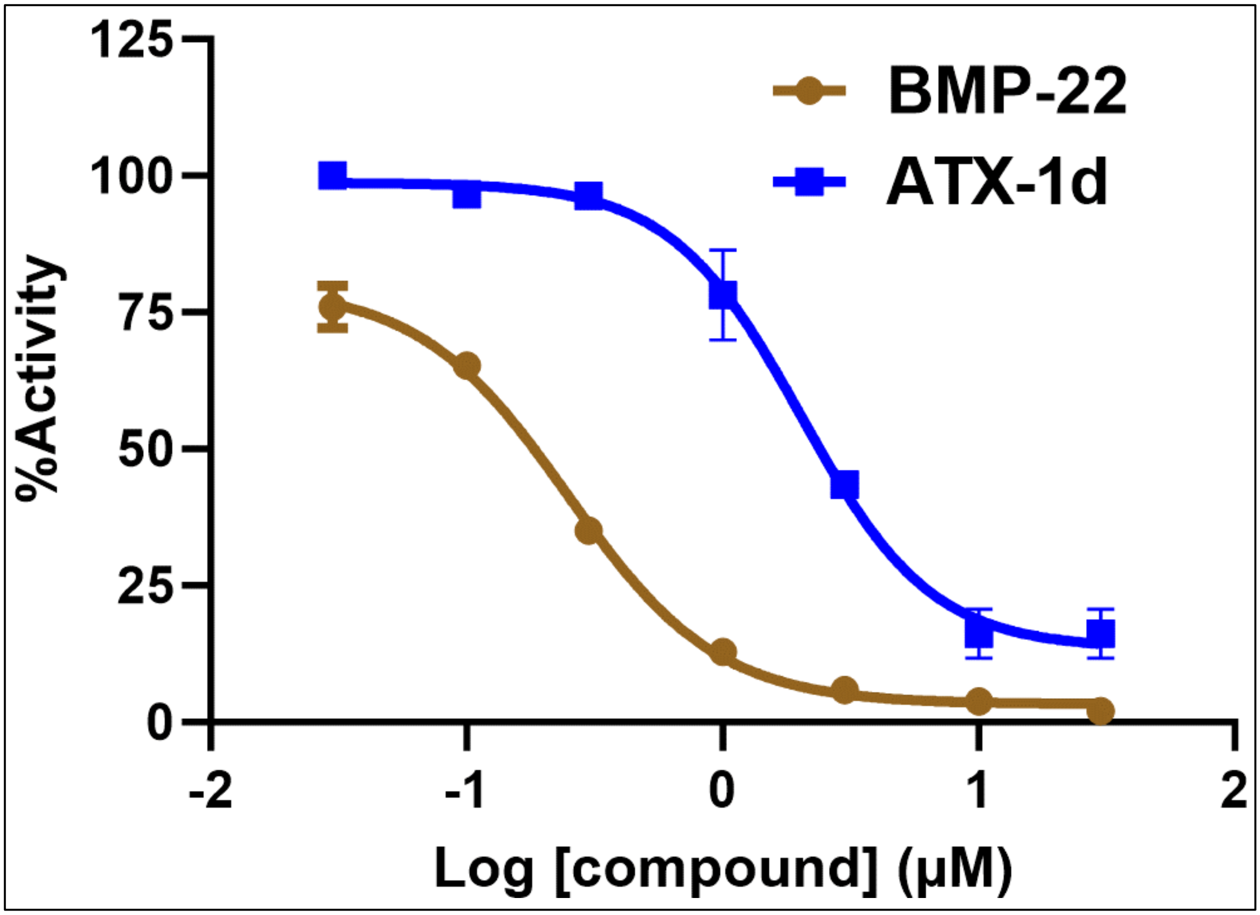

Figure S2: A second replicate of the in vitro autotaxin enzyme inhibition assay was conducted. The average IC_{50} values obtained from the biological duplicates were 1.8 ± 0.3 μM for ATX-1d and 0.2 ± 0.1 μM for BMP-22, as mentioned in the main text.

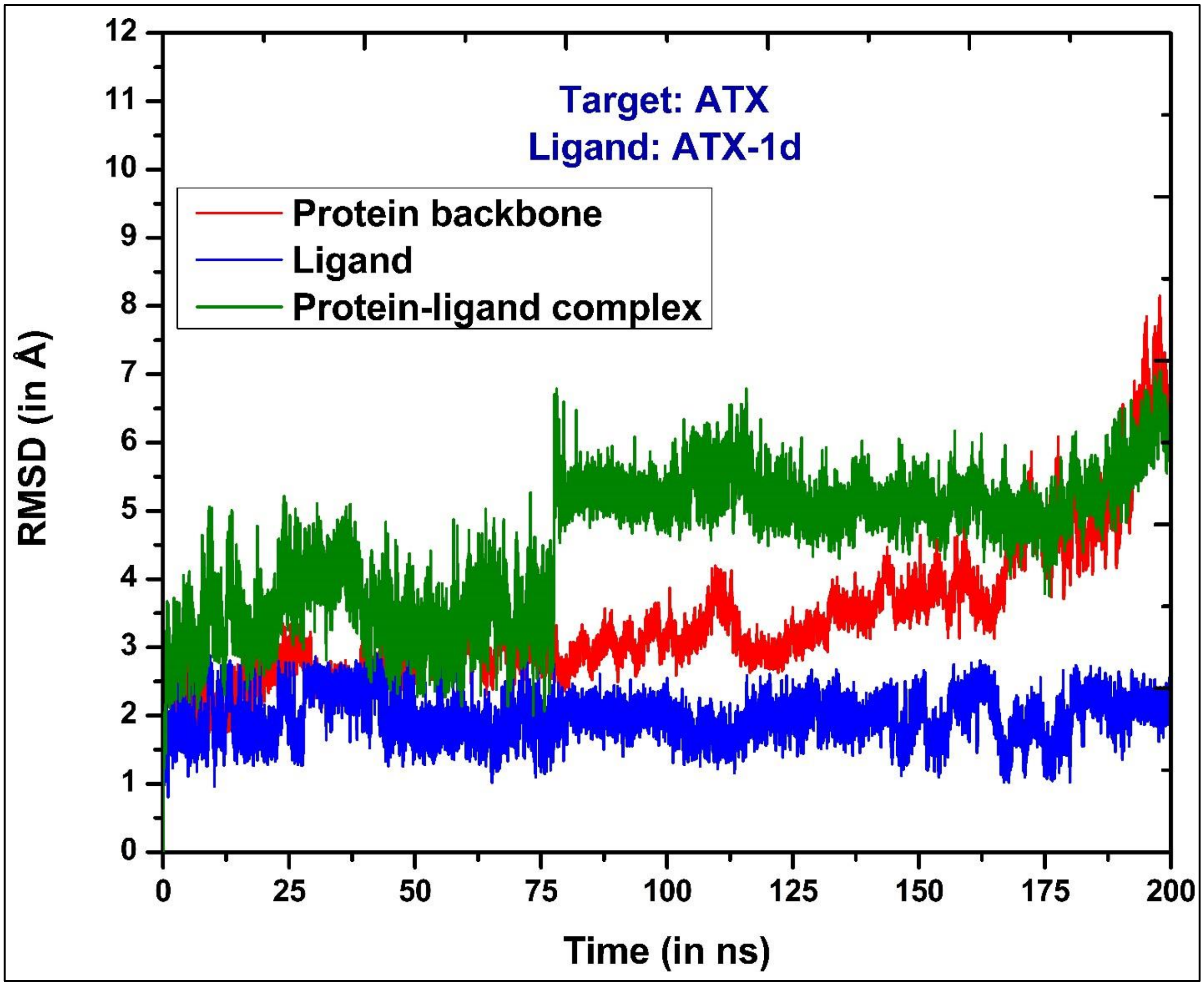

Figure S3: RMSD plot for the ATX-1d-ATX complex obtained through the 200 ns MD simulation.

Table S2: Binding free energy of ATX-1d calculated using the MM-GBSA method for the last 100 ns of the 200 ns MD simulation.

Compound vs. Target	van der Waals		Electrostatics		Polar Solvation		Non-Polar Solv.		TOTAL	
	Avg.	Std. Err. of Mean	Avg.	Std. Err. of Mean	Avg.	Std. Err. of Mean	Avg.	Std. Err. of Mean	Avg.	Std. Err. of Mean
ATX-1d vs. ATX	-44.59	0.05	-11.44	0.05	32.30	0.05	-6.11	0.01	-29.84	0.05

Table S3: Results of the SAPT0 calculations for the SKV (native ligand, PDB ID: 6W35)-ATX complex.

Residues	Electrostatics (in kcal/mol)	Exchange (in kcal/mol)	Induction (in kcal/mol)	Dispersion (in kcal/mol)	Total SAPT0 (in kcal/mol)
LEU_244	-0.51362912	0.0000133	-0.03465525	-0.20027825	-0.74854932

SER_82	0.5711108	4.6243372	-0.58220738	-2.84760474	1.76563588
PHE_211	-0.24579526	2.35711674	-0.4955449	-4.81069498	-3.1949184
TRP_261	-1.20445364	6.07908056	-0.77245323	-8.61152581	-4.50935212
TYR_215	-2.61828492	3.07595347	-0.9864936	-5.81277176	-6.34159681
ASN_231	-0.34825043	-0.00000718	-0.01440844	-0.04943876	-0.41210481
THR_210	0.43690158	0.0002091	-0.01593241	-0.19125099	0.22992728
SER_170	-0.73383312	0.90250365	-0.22317205	-1.49393978	-1.5484413
TYR_307	-0.22292421	0.3164695	-0.09210449	-1.27545492	-1.27401412
LEU_214	-1.76259431	5.81814373	-1.07768174	-8.01541237	-5.03754469
ALA_305	0.30214659	0.61221963	-0.10726856	-1.16425124	-0.35715358
PHE_274	-3.97088328	4.22002288	-0.91097252	-6.33874482	-7.00057774
PHE_275	-4.1467281	9.2844323	-1.3823188	-11.18167398	-7.42628858
LEU_79	-0.41570981	3.02172712	-0.45953865	-3.69590434	-1.54942568
LYS_249	2.8771794	2.75879512	-3.40377315	-4.34880373	-2.11660236
PHE_250	-7.12806797	11.07371316	-2.63842802	-8.73654967	-7.4293325
TYR_83	0.23882933	1.97034323	-0.66371545	-3.43941563	-1.89395852
HIS_252	-1.57822137	2.35544618	-0.387831	-3.10771007	-2.71831626
TRP_255	-8.06998014	14.62116976	-2.32813186	-15.61792282	-11.39486506
ILE_168	-2.35017823	4.43682093	-0.54232477	-1.99916296	-0.45484503
ALA_218	-0.40164195	2.33635993	-0.37955745	-2.64179048	-1.08662995
LEU_214	-1.74731907	3.42937611	-0.28117189	-2.70195291	-1.30106776
**Total**	**-33.03232723**	**83.29424642**	**-17.77968561**	**-98.28225501**	**-65.80002143**

Table S4: Results of the SAPT0 calculations for the ATX-1d-ATX complex.

Residues	Electrostatics (in kcal/mol)	Exchange (in kcal/mol)	Induction (in kcal/mol)	Dispersion (in kcal/mol)	Total SAPT0 (in kcal/mol)
LEU_244	-1.40653792	2.9848984	-0.54329581	-4.54093476	-3.50587009
SER_82	-0.97788262	1.26006161	-0.28520017	-1.73300208	-1.73602326
PHE_211	-3.33007837	6.06573608	-0.99006888	-9.35105176	-7.60546293
TRP_261	0.16344757	0.00009637	-0.01459478	-0.19620693	-0.04725777
TYR_215	-0.03048442	1.28863389	-0.19891648	-2.61089658	-1.55166359
ASN_231	-0.19717761	-0.00005729	-0.01877983	-0.12030549	-0.33632022
THR_210	-0.20379138	0.10884682	-0.09706061	-0.95875912	-1.15076429
SER_170	-0.02975707	-0.00000233	-0.0022096	-0.02532357	-0.05729257
TYR_307	-0.25037932	0.71209333	-0.14730383	-2.30343828	-1.9890281
LEU_214	-0.0825461	1.11228228	-0.19383785	-2.28016137	-1.44426304
ALA_305	-0.01135899	-0.00000017	-0.00178154	-0.01700991	-0.03015061
PHE_274	0.1164978	-0.00008607	-0.03083938	-0.33114816	-0.24557581
PHE_275	-5.33508004	10.59961558	-1.95079193	-11.04883348	-7.73508987
LEU_79	0.06969068	0.38530968	-0.04145887	-0.96003275	-0.54649126
LYS_249	-11.84001823	8.37679297	-6.35134428	-7.0190447	-16.83361424

PHE_250	0.23748682	0.00175884	-0.0192196	-0.36832294	-0.14829688
TYR_83	0.19802493	-0.00010204	-0.00671926	-0.17362996	0.01757367
HIS_252	-0.29156693	-0.00002733	-0.00867775	-0.08540559	-0.3856776
TRP_255	-0.20717571	1.58339561	-0.23961238	-4.15798781	-3.02138029
ILE_168	-0.03649502	0	-0.00092751	-0.01180188	-0.04922441
ALA_218	0.09428167	-0.00000302	-0.00250141	-0.02928226	0.06249498
LEU_214	-0.0825461	1.11228228	-0.19383785	-2.28016137	-1.44426304
**Total**	**-23.43344636**	**35.59152549**	**-11.3389796**	**-50.60274075**	**-49.78364122**

Table S5: Evaluation of pharmacokinetics, drug-likeness, and medicinal chemistry friendliness predictions of ATX-1d through the *SwissADME* webserver.

**Molecule Name**	**ATX-1d**
**Physicochemical properties**	
Canonical SMILES	CCN1C(=O)/C(=C(/c2ccc(cc2)C(C)(C)C)\C(=O)OC)/C(C1=O)(c1ccccn1)C1CC(=O)N(C1=O)CC
Formula	C30H33N3O6
MW	531.6
#Heavy atoms	39
#Aromatic heavy atoms	12
Fraction Csp3	0.4
#Rotatable bonds	8
#H-bond acceptors	7
#H-bond donors	0
MR	151.94
TPSA	113.95
**Lipophilicity**	
iLOGP	3.94
XLOGP3	2.76
WLOGP	2.27
MLOGP	2.19
Silicos-IT Log P	4.53

Consensus Log P	3.14
**Water solubility**	
ESOL Log S	-4.57
ESOL Solubility (mg/ml)	1.42e-02
ESOL Solubility (mol/l)	2.66e-05
ESOL Class	Moderately soluble
Ali Log S	-4.81
Ali Solubility (mg/ml)	8.27e-03
Ali Solubility (mol/l)	1.56e-05
Ali Class	Moderately soluble
Silicos-IT LogSw	-7.43
Silicos-IT Solubility (mg/ml)	1.97e-05
Silicos-IT Solubility (mol/l)	3.70e-08
Silicos-IT class	Poorly soluble
**Pharmacokinetics**	
GI absorption	High
BBB permeant	No
Pgp substrate	No
CYP1A2 inhibitor	No
CYP2C19 inhibitor	No
CYP2C9 inhibitor	Yes
CYP2D6 inhibitor	No
CYP3A4 inhibitor	Yes
log Kp (cm/s)	-7.58
**Druglikeness**	
Lipinski #violations	1
Ghose #violations	3
Veber #violations	0
Egan #violations	0

Muegge #violations	0
Bioavailability Score	0.55
**Medicinal Chemistry**	
PAINS #alerts	0
Brenk #alerts	2
Leadlikeness #violations	2
Synthetic Accessibility	5.14

Table S6: Prediction of ADMET properties of ATX-1d through the *pkCSM* webserver based on graph-based signatures.

Property	Model Name	Predicted value	Unit
**Absorption**	Water solubility	**-5.827**	Numeric (log mol/L)
**Absorption**	Caco2 permeability	**0.954**	Numeric (log Papp in 10^{-6} cm/s)
**Absorption**	Intestinal absorption (human)	**88.412**	Numeric (% Absorbed)
**Absorption**	Skin Permeability	**-2.77**	Numeric (log Kp)
**Absorption**	P-glycoprotein substrate	**Yes**	Categorical (Yes/No)
**Absorption**	P-glycoprotein I inhibitor	**Yes**	Categorical (Yes/No)
**Absorption**	P-glycoprotein II inhibitor	**Yes**	Categorical (Yes/No)
**Distribution**	VDss (human)	**-0.147**	Numeric (log L/kg)
**Distribution**	Fraction unbound (human)	**0**	Numeric (Fu)
**Distribution**	BBB permeability	**-0.451**	Numeric (log BB)
**Distribution**	CNS permeability	**-3.4**	Numeric (log PS)
**Metabolism**	CYP2D6 substrate	**No**	Categorical (Yes/No)
**Metabolism**	CYP3A4 substrate	**Yes**	Categorical (Yes/No)
**Metabolism**	CYP1A2 inhibitor	**No**	Categorical (Yes/No)
**Metabolism**	CYP2C19 inhibitor	**No**	Categorical (Yes/No)
**Metabolism**	CYP2C9 inhibitor	**No**	Categorical (Yes/No)
**Metabolism**	CYP2D6 inhibitor	**No**	Categorical (Yes/No)

**Metabolism**	CYP3A4 inhibitor	**Yes**	Categorical (Yes/No)
**Excretion**	Total Clearance	**0.623**	Numeric (log ml/min/kg)
**Excretion**	Renal OCT2 substrate	**No**	Categorical (Yes/No)
**Toxicity**	AMES toxicity	**No**	Categorical (Yes/No)
**Toxicity**	Max. tolerated dose (human)	**-0.12**	Numeric (log mg/kg/day)
**Toxicity**	hERG I inhibitor	**No**	Categorical (Yes/No)
**Toxicity**	hERG II inhibitor	**Yes**	Categorical (Yes/No)
**Toxicity**	Oral Rat Acute Toxicity (LD50)	**2.934**	Numeric (mol/kg)
**Toxicity**	Oral Rat Chronic Toxicity (LOAEL)	**1.978**	Numeric (log mg/kg_bw/day)
**Toxicity**	Hepatotoxicity	**Yes**	Categorical (Yes/No)
**Toxicity**	Skin Sensitisation	**No**	Categorical (Yes/No)
**Toxicity**	T.Pyriformis toxicity	**0.29**	Numeric (log ug/L)
**Toxicity**	Minnow toxicity	**3.175**	Numeric (log mM)

Table S7: Results of the SwissTargetPrediction webserver.

SwissTargetPrediction

Target	Common name	Uniprot ID	ChEMBL ID	Target Class	Probability*	Known actives (3D/2D)
Angiotensin-converting enzyme (by homology)	ACE	P12821	CHEMBL1808	Protease	0.0956237870388	4 / 0
Gamma-secretase	PSEN2<br>PSENEN<br>NCSTN<br>APH1A<br>PSEN1<br>APH1B	P49810<br>Q9NZ42<br>Q92542<br>Q96BI3<br>P49768<br>Q8WW43	CHEMBL2094135	Protease	0.0956237870388	287 / 0
Bromodomain-containing protein 4	BRD4	O60885	CHEMBL1163125	Reader	0.0956237870388	185 / 0
Bromodomain-containing protein 2	BRD2	P25440	CHEMBL1293289	Reader	0.0956237870388	93 / 0
Bromodomain-containing protein 3	BRD3	Q15059	CHEMBL1795186	Reader	0.0956237870388	98 / 0
Thrombin and coagulation factor X	F10	P00742	CHEMBL244	Protease	0.0956237870388	283 / 0
Calpain 1	CAPN1	P07384	CHEMBL3891	Protease	0.0956237870388	172 / 0
Tyrosine-protein kinase JAK3	JAK3	P52333	CHEMBL2148	Kinase	0.0956237870388	331 / 0
Tyrosine-protein kinase JAK1	JAK1	P23458	CHEMBL2835	Kinase	0.0956237870388	217 / 0
Tyrosine-protein kinase JAK2	JAK2	O60674	CHEMBL2971	Kinase	0.0956237870388	398 / 0
Complement factor D	CFD	P00746	CHEMBL2176771	Protease	0.0956237870388	191 / 0
Histone deacetylase 6	HDAC6	Q9UBN7	CHEMBL1865	Eraser	0.0956237870388	86 / 0
Histone deacetylase 2	HDAC2	Q92769	CHEMBL1937	Eraser	0.0956237870388	70 / 0
Histone deacetylase 3/ Nuclear receptor corepressor 2 (HDAC3/NCoR2)	NCOR2<br>HDAC3	Q9Y618<br>O15379	CHEMBL2111363	Eraser	0.0956237870388	15 / 0
Histone deacetylase 1	HDAC1	Q13547	CHEMBL325	Eraser	0.0956237870388	191 / 0
Histone deacetylase 4	HDAC4	P56524	CHEMBL3524	Eraser	0.0956237870388	16 / 0
Phosphodiesterase 10A	PDE10A	Q9Y233	CHEMBL4409	Phosphodiesterase	0.0956237870388	1109 / 0
Translocator protein (by homology)	TSPO	P30536	CHEMBL5742	Membrane receptor	0.0956237870388	324 / 0
c-Jun N-terminal kinase 1	MAPK8	P45983	CHEMBL2276	Kinase	0.0956237870388	335 / 0
Epoxide hydratase	EPHX2	P34913	CHEMBL2409	Protease	0.0956237870388	267 / 0

Target	Common name	Uniprot ID	ChEMBL ID	Target Class	Probability*	Known actives (3D/2D)
Cathepsin K	CTSK	P43235	CHEMBL268	Protease	0.0956237870388	404 / 0
Cathepsin (B and K)	CTSB	P07858	CHEMBL4072	Protease	0.0956237870388	219 / 0
Legumain	LGMN	Q99538	CHEMBL4244	Protease	0.0956237870388	23 / 0
Sonic hedgehog protein	SHH	Q15465	CHEMBL5602	Unclassified protein	0.0956237870388	19 / 0
Cathepsin S	CTSS	P25774	CHEMBL2954	Protease	0.0956237870388	310 / 0
Matrix metalloproteinase 9	MMP9	P14780	CHEMBL321	Protease	0.0956237870388	113 / 0
Cathepsin L	CTSL	P07711	CHEMBL3837	Protease	0.0956237870388	229 / 0
Histone deacetylase 3	HDAC3	O15379	CHEMBL1829	Eraser	0.0956237870388	39 / 0
Thrombin	F2	P00734	CHEMBL204	Protease	0.0956237870388	152 / 0
Protein farnesyltransferase	FNTA FNTB	P49354 P49356	CHEMBL2094108	Enzyme	0.0956237870388	431 / 0
c-Jun N-terminal kinase 2	MAPK9	P45984	CHEMBL4179	Kinase	0.0956237870388	137 / 0
Calpain 1	CAPN1 CAPNS1	P07384 P04632	CHEMBL2111357	Protease	0.0956237870388	22 / 0
Dopamine D2 receptor	DRD2	P14416	CHEMBL217	Family A G protein-coupled receptor	0.0956237870388	267 / 0
Dopamine D4 receptor	DRD4	P21917	CHEMBL219	Family A G protein-coupled receptor	0.0956237870388	159 / 0
Fibroblast activation protein alpha	FAP	Q12884	CHEMBL4683	Protease	0.0956237870388	68 / 0
Nerve growth factor receptor Trk-A	NTRK1	P04629	CHEMBL2815	Kinase	0.0956237870388	165 / 0
JAK3/JAK1	JAK3 JAK1	P52333 P23458	CHEMBL3038491	Kinase	0.0956237870388	24 / 0
Cannabinoid receptor 1	CNR1	P21554	CHEMBL218	Family A G protein-coupled receptor	0.0956237870388	810 / 0
Phosphodiesterase 2A	PDE2A	O00408	CHEMBL2652	Phosphodiesterase	0.0956237870388	116 / 0
Sodium channel protein type IX alpha subunit	SCN9A	Q15858	CHEMBL4296	Voltage-gated ion channel	0.0956237870388	130 / 0
Poly [ADP-ribose] polymerase-1	PARP1	P09874	CHEMBL3105	Enzyme	0.0956237870388	227 / 0
Vanilloid receptor	TRPV1	Q8NER1	CHEMBL4794	Voltage-gated ion channel	0.0956237870388	366 / 0
Poly [ADP-ribose] polymerase 3	PARP3	Q9Y6F1	CHEMBL5083	Enzyme	0.0956237870388	7 / 0
Metabotropic glutamate receptor 5	GRM5	P41594	CHEMBL3227	Family C G protein-coupled receptor	0.0956237870388	623 / 0
Glycine transporter 1	SLC6A9	P48067	CHEMBL2337	Electrochemical transporter	0.0956237870388	101 / 0
Glycine transporter 2	SLC6A5	Q9Y345	CHEMBL3060	Electrochemical transporter	0.0956237870388	6 / 0

Target	Common name	Uniprot ID	ChEMBL ID	Target Class	Probability*	Known actives (3D/2D)
Formyl peptide receptor 1	FPR1	P21462	CHEMBL3359	Family A G protein-coupled receptor	0.0956237870388	14 / 0
Heat shock protein HSP 90-alpha	HSP90AA1	P07900	CHEMBL3880	Other cytosolic protein	0.0956237870388	81 / 0
Lipoxin A4 receptor	FPR2	P25090	CHEMBL4227	Family A G protein-coupled receptor	0.0956237870388	23 / 0
p53-binding protein Mdm-2	MDM2	Q00987	CHEMBL5023	Other nuclear protein	0.0956237870388	106 / 0
Delta opioid receptor	OPRD1	P41143	CHEMBL236	Family A G protein-coupled receptor	0.0956237870388	49 / 0
FK506-binding protein 1A	FKBP1A	P62942	CHEMBL1902	Isomerase	0.0956237870388	125 / 0
Sodium channel protein type V alpha subunit	SCN5A	Q14524	CHEMBL1980	Voltage-gated ion channel	0.0956237870388	34 / 0
HERG	KCNH2	Q12809	CHEMBL240	Voltage-gated ion channel	0.0956237870388	189 / 0
MAP kinase p38 alpha	MAPK14	Q16539	CHEMBL260	Kinase	0.0956237870388	697 / 0
Isocitrate dehydrogenase [NADP] cytoplasmic	IDH1	O75874	CHEMBL2007625	Enzyme	0.0956237870388	116 / 0
Cholecystokinin B receptor	CCKBR	P32239	CHEMBL298	Family A G protein-coupled receptor	0.0956237870388	410 / 0
Neurokinin 3 receptor	TACR3	P29371	CHEMBL4429	Family A G protein-coupled receptor	0.0956237870388	134 / 0
Orexin receptor 2	HCRTR2	O43614	CHEMBL4792	Family A G protein-coupled receptor	0.0956237870388	945 / 0
Orexin receptor 1	HCRTR1	O43613	CHEMBL5113	Family A G protein-coupled receptor	0.0956237870388	788 / 0
Serine/threonine-protein kinase B-raf	BRAF	P15056	CHEMBL5145	Kinase	0.0956237870388	105 / 0
HLA class II histocompatibility antigen DRB1-1	HLA-DRB1	P04229	CHEMBL1943	Surface antigen	0.0956237870388	16 / 0
Tryptophan 2,3-dioxygenase	TDO2	P48775	CHEMBL2140	Enzyme	0.0956237870388	13 / 0
Leukocyte elastase	ELANE	P08246	CHEMBL248	Protease	0.0956237870388	149 / 0
Histone deacetylase 8	HDAC8	Q9BY41	CHEMBL3192	Eraser	0.0956237870388	33 / 0
Neuropeptide Y receptor type 5	NPY5R	Q15761	CHEMBL4561	Family A G protein-coupled receptor	0.0956237870388	231 / 0
Kinesin-1 heavy chain/ Tyrosine-protein kinase receptor RET	RET	P07949	CHEMBL2041	Kinase	0.0956237870388	61 / 0
Neurokinin 1 receptor	TACR1	P25103	CHEMBL249	Family A G protein-coupled receptor	0.0956237870388	157 / 0
Kir3.1/Kir3.4	KCNJ5 KCNJ3	P48544 P48549	CHEMBL3038488	Voltage-gated ion channel	0.0956237870388	28 / 0
Kir3.1/Kir3.2	KCNJ6 KCNJ3	P48051 P48549	CHEMBL3038489	Voltage-gated ion channel	0.0956237870388	31 / 0

Target	Common name	Uniprot ID	ChEMBL ID	Target Class	Probability*	Known actives (3D/2D)
Ghrelin receptor	GHSR	Q92847	CHEMBL4616	Family A G protein-coupled receptor	0.0956237870388	30 / 0
TNF-alpha	TNF	P01375	CHEMBL1825	Secreted protein	0.0956237870388	5 / 0
Adenosine A3 receptor	ADORA3	P0DMS8	CHEMBL256	Family A G protein-coupled receptor	0.0956237870388	298 / 0
Receptor-interacting serine/ threonine-protein kinase 1	RIPK1	Q13546	CHEMBL5464	Kinase	0.0956237870388	13 / 0
Kappa Opioid receptor	OPRK1	P41145	CHEMBL237	Family A G protein-coupled receptor	0.0956237870388	58 / 0
Mannose-6-phosphate isomerase	MPI	P34949	CHEMBL2758	Isomerase	0.0956237870388	13 / 0
Chymase	CMA1	P23946	CHEMBL4068	Protease	0.0956237870388	88 / 0
Cytochrome P450 1A1 (by homology)	CYP1A1	P04798	CHEMBL2231	Cytochrome P450	0.0956237870388	8 / 0
Calpain 2	CAPN2	P17655	CHEMBL2382	Protease	0.0956237870388	39 / 0
Cannabinoid receptor 2	CNR2	P34972	CHEMBL253	Family A G protein-coupled receptor	0.0956237870388	738 / 0
JAK2/JAK1	JAK1 JAK2	P23458 O60674	CHEMBL3038492	Kinase	0.0956237870388	4 / 0
DNA-directed RNA polymerase I subunit RPA1	POLR1A	O95602	CHEMBL3286067	Enzyme	0.0956237870388	2 / 0
Hepatocyte growth factor receptor	MET	P08581	CHEMBL3717	Kinase	0.0956237870388	439 / 0
Dual-specificity tyrosine-phosphorylation regulated kinase 1A	DYRK1A	Q13627	CHEMBL2292	Kinase	0.0956237870388	74 / 0
3-phosphoinositide dependent protein kinase-1	PDPK1	O15530	CHEMBL2534	Kinase	0.0956237870388	63 / 0
Phosphodiesterase 4B	PDE4B	Q07343	CHEMBL275	Phosphodiesterase	0.0956237870388	221 / 0
PI3-kinase p110-alpha subunit (by homology)	PIK3CA	P42336	CHEMBL4005	Enzyme	0.0956237870388	354 / 0
Nitric oxide synthase, inducible	NOS2	P35228	CHEMBL4481	Enzyme	0.0956237870388	133 / 0
Leucine-rich repeat serine/threonine-protein kinase 2	LRRK2	Q5S007	CHEMBL1075104	Kinase	0.0956237870388	98 / 0
Phosphodiesterase 7A	PDE7A	Q13946	CHEMBL3012	Phosphodiesterase	0.0956237870388	95 / 0
Melanin-concentrating hormone receptor 1	MCHR1	Q99705	CHEMBL344	Family A G protein-coupled receptor	0.0956237870388	29 / 0
Tyrosine-protein kinase TYK2	TYK2	P29597	CHEMBL3553	Kinase	0.0956237870388	88 / 0

Target	Common name	Uniprot ID	ChEMBL ID	Target Class	Probability*	Known actives (3D/2D)
Phosphodiesterase 5A	PDE5A	O76074	CHEMBL1827	Phosphodiesterase	0.0956237870388	295 / 0
Plasma kallikrein	KLKB1	P03952	CHEMBL2000	Protease	0.0956237870388	9 / 0
Neuronal acetylcholine receptor protein alpha-7 subunit	CHRNA7	P36544	CHEMBL2492	Ligand-gated ion channel	0.0956237870388	14 / 0
c-Jun N-terminal kinase 3	MAPK10	P53779	CHEMBL2637	Kinase	0.0956237870388	204 / 0
Coagulation factor XI	F11	P03951	CHEMBL2820	Protease	0.0956237870388	15 / 0
Bradykinin B1 receptor	BDKRB1	P46663	CHEMBL4308	Family A G protein-coupled receptor	0.0956237870388	40 / 0
Cytochrome P450 26A1	CYP26A1	O43174	CHEMBL5141	Cytochrome P450	0.0956237870388	14 / 0
Glutamate NMDA receptor; GRIN1/ GRIN2A	GRIN2A GRIN1	Q12879 Q05586	CHEMBL1907604	Ligand-gated ion channel	0.0956237870388	15 / 0
